# Procalcitonin-guided antibiotic therapy may shorten length of treatment and may improve survival—a systematic review and meta-analysis

**DOI:** 10.1186/s13054-023-04677-2

**Published:** 2023-10-13

**Authors:** Márton Papp, Nikolett Kiss, Máté Baka, Domonkos Trásy, László Zubek, Péter Fehérvári, Andrea Harnos, Caner Turan, Péter Hegyi, Zsolt Molnár

**Affiliations:** 1 Centre for Translational Medicine, Semmelweis University, Üllői Út 26, 1082 Budapest, Hungary; 2 Department of Anesthesiology and Intensive Therapy, Saint John’s Hospital, Budapest, Hungary; 3 Department of Anesthesiology and Intensive Therapy, Heart and Vascular Center, Semmelweis University, Budapest, Hungary; 4 Department of Anesthesiology and Intensive Therapy, Semmelweis University, Budapest, Hungary; 5 Department of Anesthesiology and Intensive Therapy, Faculty of Medicine, Poznan University of Medical Sciences, Poznan, Poland; 6 Department of Biostatistics, University of Veterinary Medicine, Budapest, Hungary; 7 Institute of Pancreatic Diseases, Semmelweis University, Budapest, Hungary; 8 Institute for Translational Medicine, Medical School, University of Pécs, Pécs, Hungary

**Keywords:** Intensive care, Procalcitonin, Antibiotic therapy, Sepsis

## Abstract

**Background:**

Appropriate antibiotic (AB) therapy remains a challenge in the intensive care unit (ICU). Procalcitonin (PCT)-guided AB stewardship could help optimize AB treatment and decrease AB-related adverse effects, but firm evidence is still lacking. Our aim was to compare the effects of PCT-guided AB therapy with standard of care (SOC) in critically ill patients.

**Methods:**

We searched databases CENTRAL, Embase and Medline. We included randomized controlled trials (RCTs) comparing PCT-guided AB therapy (PCT group) with SOC reporting on length of AB therapy, mortality, recurrent and secondary infection, ICU length of stay (LOS), hospital LOS or healthcare costs. Due to recent changes in sepsis definitions, subgroup analyses were performed in studies applying the Sepsis-3 definition. In the statistical analysis, a random-effects model was used to pool effect sizes.

**Results:**

We included 26 RCTs (*n* = 9048 patients) in the quantitative analysis. In comparison with SOC, length of AB therapy was significantly shorter in the PCT group (MD − 1.79 days, 95% CI: -2.65, − 0.92) and was associated with a significantly lower 28-day mortality (OR 0.84, 95% CI: 0.74, 0.95). In Sepsis-3 patients, mortality benefit was more pronounced (OR 0.46 95% CI: 0.27, 0.79). Odds of recurrent infection were significantly higher in the PCT group (OR 1.36, 95% CI: 1.10, 1.68), but there was no significant difference in the odds of secondary infection (OR 0.81, 95% CI: 0.54, 1.21), ICU and hospital length of stay (MD − 0.67 days 95% CI: − 1.76, 0.41 and MD − 1.23 days, 95% CI: − 3.13, 0.67, respectively).

**Conclusions:**

PCT-guided AB therapy may be associated with reduced AB use, lower 28-day mortality but higher infection recurrence, with similar ICU and hospital length of stay. Our results render the need for better designed studies investigating the role of PCT-guided AB stewardship in critically ill patients.

**Graphical abstract:**

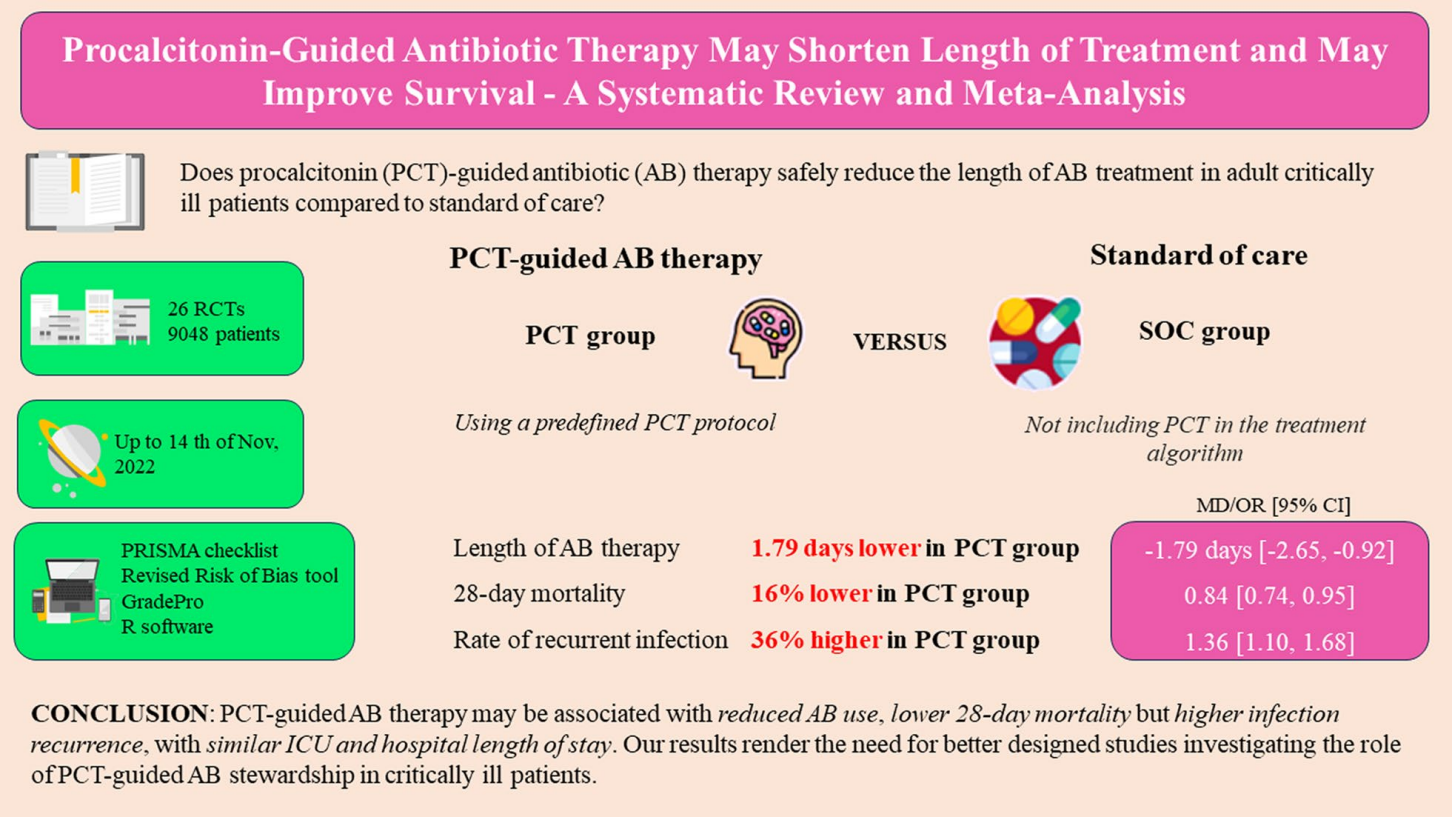

**Supplementary Information:**

The online version contains supplementary material available at 10.1186/s13054-023-04677-2.

## Introduction

Inappropriate use of antibiotics (ABs) has serious adverse effects. As a result, antibiotic resistance is emerging, causing approximately 700,000 deaths worldwide in 2014 and is predicted to be the leading cause of death worldwide by 2050—accounting for 10 million deaths per year [1]. Critically ill patients in ICU are at high risk of becoming infected with multidrug-resistant organisms (MDRO) due to their acquired immune deficiency, resulting in unacceptably high morbidity and mortality [2].

In general, more than 50% of critically ill patients are considered as infected. Infection and related sepsis can more than double ICU mortality [3]. However, less than 60% of critically ill patients with an initial diagnosis of sepsis are confirmed to be infected [4]. Despite the known challenges in the differential diagnosis of infection and sepsis, there is an urgent constraint to administer ABs shortly after the onset of sepsis and septic shock [5]. This strategy may inevitably result in unnecessary AB therapy, thus increasing the chance of harm and costs associated with AB treatment.

Procalcitonin (PCT) is one of the most studied inflammatory biomarkers [6] and can distinguish bacterial infections from viral infections in critically ill patients [7, 8]. There is growing evidence that PCT-guided AB therapy can safely reduce antimicrobial consumption—by reducing the number of unnecessary or excessively long therapies. The results of a large, individual patient data meta-analysis in 2017 support the use of PCT in the management of AB stewardship in acute respiratory infections in a variety of clinical settings [9]. However, the evidence is less convincing in other types of infection and sepsis.

The PRORATA study was the first big, multicenter RCT to demonstrate the efficacy and non-inferiority of AB management guided by a predefined PCT protocol in septic critically ill patients [10]. Subsequent trials conducted in ICUs used an approach identical with or similar to PRORATA, but PCT levels for starting and stopping thresholds varied, patient populations were also heterogeneous with medical, surgical, or mixed populations treated for different types of infections, and therefore the overall interpretation and implementation of PCT-guided AB therapy in ICU setting remains challenging. Moreover, with the implementation of the new Sepsis-3 definition [11], study inclusion criteria for sepsis and septic shock have also changed in the most recent clinical trials as compared to the definitions previously used for decades [12, 13]. An updated comprehensive analysis of PCT stewardship in ICU setting, including Sepsis-3 patients, was lacking.

Therefore, we aimed to perform a systematic review and meta-analysis of randomized controlled trials (RCTs) that investigated the effects of PCT-guided AB therapy compared to standard of care (SOC) in critically ill patients.

## Methods

We report our systematic review and meta-analysis based on the recommendations of the PRISMA 2020 guideline [14] (see Additional file 1: Table S1), while we followed the Cochrane Handbook [15]. The protocol of the study was registered on PROSPERO (registration number CRD42022374605), and we adhered to it except for one additional outcome measure (rate of secondary infection) and two subgroup analyses (PCT protocol and patient population).

## Eligibility criteria

Applying the PICO (Population, Intervention, Comparator, Outcome) framework, we included RCTs that were conducted in P: adult patients with known or suspected infection treated with antibiotics; I: PCT-guided AB therapy; C: SOC (without PCT use); and they provided data on either of the following, O: length of AB therapy, mortality, rate of recurrent infection (clinically confirmed infection in the same location caused by the same pathogen as the primary one), rate of secondary infection (clinically confirmed infection caused by an organism different from the primary one), length of ICU stay, length of hospital stay and healthcare costs. RCTs conducted in the ICU were included in the quantitative, those conducted in other clinical settings, were included in the qualitative analysis.

## Information sources and search strategy

Our systematic search was conducted in three main databases—CENTRAL, Embase and Medline—on November 14, 2022. We used the following search key in all databases: (sepsis OR septic OR infection) AND (PCT OR procalcitonin) AND (antibiotic* OR antimicrobial OR anti-microbial). Conference papers were excluded.

## Selection process and data extraction

Selection was performed by two independent review authors (M.P. and N.K.) using a reference management software (EndNote 20, Clarivate Analytics). After automatic and manual duplicate removal, reviewers screened titles and abstracts, then full texts against predefined eligibility criteria. Data were collected independently by two authors (M.P. and M.B.) on a standardized data extraction sheet. We used Google translate for an article in Chinese [16]. The following data were extracted in addition to the previously mentioned outcomes: digital object identifier, first author, publication year, countries, centers, study period, study population, sepsis definition, age, gender, PCT protocol, protocol adherence, appropriateness of AB therapy, and exclusion criteria.

## Subgroup analysis

We planned to perform subgroup analyses to reduce heterogeneity according to the applied sepsis definitions (Sepsis-1 [13], 2 [12] and 3 [11]), PCT protocol (liberal—stop AB if PCT reduced > 80% of the peak value or < 0.5 ng/mL; and conservative—stop AB if PCT reduced > 90% of peak value or < 0.1–0.25 ng/mL or < 1 ng/mL for 3 days) and patient population (medical, surgical and mixed). We considered ventilator-associated pneumonia (VAP) as pulmonary sepsis.

## Risk of bias assessment and evidence level

Three authors (M.P., M.B. and D.T.) performed the risk of bias assessment independently using the revised Cochrane risk-of-bias tool for randomized trials (RoB 2) [17] and GRADE Pro [18] to assess the quality of evidence, with disagreements resolved by another author (C.T.).

## Synthesis methods

At least three studies had to be included to perform a meta-analysis. As we assumed considerable between-study heterogeneity in all cases, a random-effects model was used to pool effect sizes.

For dichotomous outcomes, odds ratio (OR) with 95% confidence interval (CI) was used to measure the effect size. Pooled OR based on raw data was calculated using the Mantel–Haenszel method [19, 20]. For continuous outcomes, the difference between means (MD) was used to measure the effect size. To calculate the pooled difference, the sample size, the mean and the corresponding standard deviation (SD) were extracted from each study. If the SD was not provided, but the standard error (SE) or confidence interval was available, we calculated the SD from it. The inverse variance weighting method was used to calculate the pooled MD.

We used a Hartung-Knapp adjustment if it resulted in a more conservative estimate than without adjustment [21, 22]. Results were considered statistically significant if the CI did not include the value zero. We summarized the findings for the meta-analysis in forest plots. Where appropriate, we reported the prediction intervals (i.e., the expected range of effects of future studies) of results. Heterogeneity was assessed using Higgins and Thompson *I*^2^ statistics [23].

All statistical analyses were performed with *R* (R Core Team 2023, v4.2.3) [24], using the *meta* (Schwarzer 2023, v6.2.1) [25] package for basic meta-analysis calculations and plots, and *dmetar* (Cuijpers, Furukawa, and Ebert 2023, v0.0.9000) [26] package for additional influential analysis calculations and plots.

When necessary and possible, model fitting parameters, and potential outlier publications were explored using different influence measures and plots (e.g., leave-one-out analysis for changes in fitted values, Bujat diagnostics values and plots) as recommended by Harrer et al. (2021) [27]. Small study publication bias was assessed by visual inspection of funnel plots and Egger's test (modified Egger’s test depends on the type of effect size measures) with 10% significance level [28].

For subgroup analysis, we used a fixed-effects “plural” model (aka. mixed-effects model). We assumed that subgroups had different τ^2^ values as we anticipated differences in the between-study heterogeneity in the subgroups, although for practical reasons, if any of the subgroup size was five or less, a common τ^2^ assumption was used [29].

## Results

### Search and selection

**Fig. 1:**
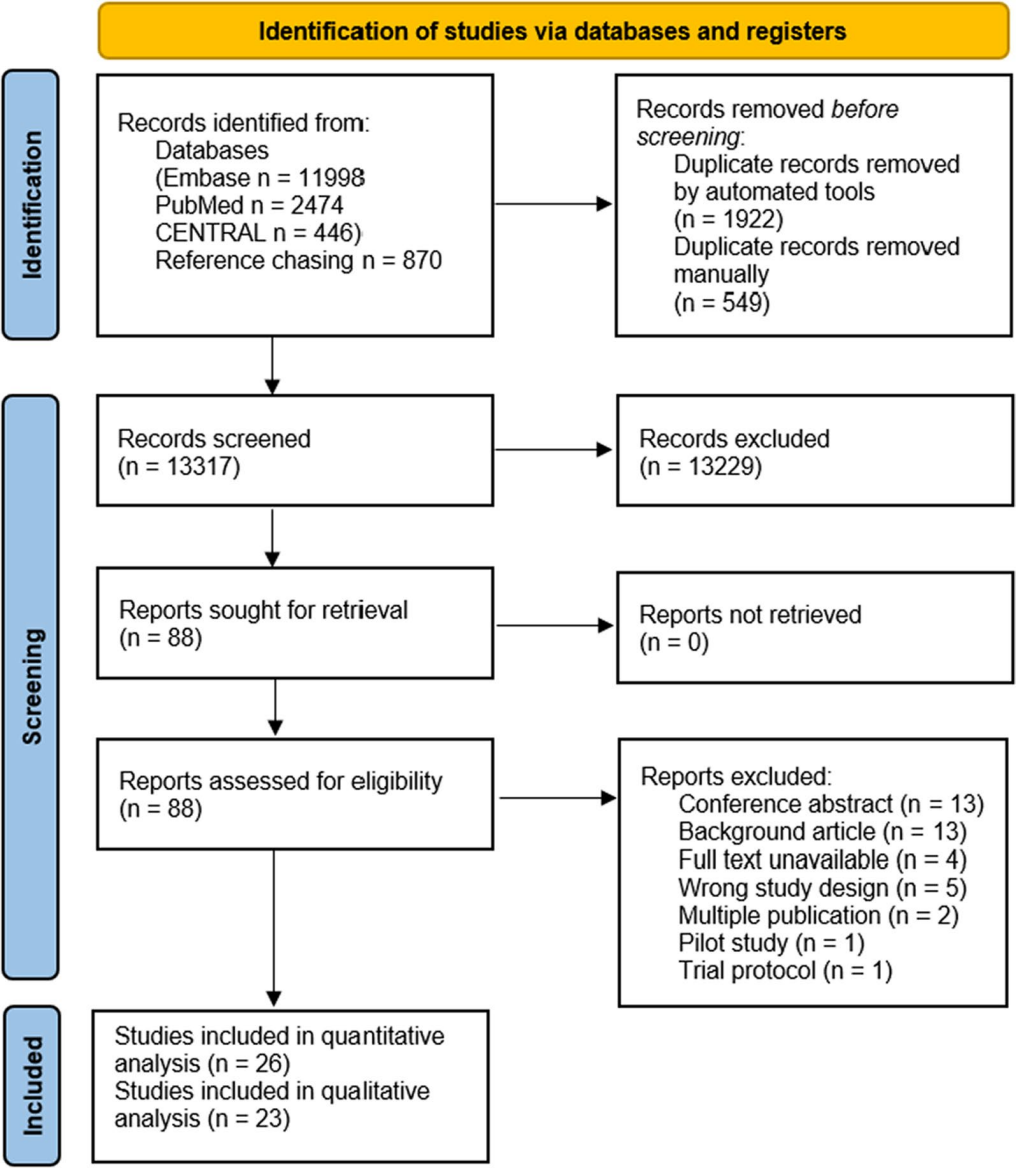
PRISMA 2020 flowchart representing the study selection process

Our systematic search resulted in 15,788 eligible articles. After the selection process, 26 articles were included in the meta-analysis [10, 16, 30–53] and 23 articles in the systematic review. The latter included those patients who were treated outside the ICU [54–76]. Figure 1 shows the PRISMA 2020 Flow diagram of the search.

## Basic characteristics of included studies

Baseline characteristics of the included studies are detailed in Table 1. Other relevant information is summarized in Additional file 1: Table S2.

**Table 1:** Characteristics of studies included

Study author	Country (centers)	Sample size (female% in PCT/control arm)	Clinical setting	Patient population	Age ^a^ in PCT/control arm
Kyriazopoulou et al., [30]	Greece (7)	256 (59/54)	ICU ^b^	Medical patients with sepsis	80 ± 10 / 78 ± 13
Ali et al., [32]	Egypt (1)	60 (43/47)	ICU	Patients with sepsis	57 ± 1 / 56 ± 2
Vishalashi et al., [31]	India (1)	90 (51/36)	ICU	Medical/surgical (41/59%) patients with sepsis	42 ± 17 / 47 ± 16
Labro et al., [33]	France (5)	159 (35/47)	ICU	Mechanically ventilated medical patients with coma	54 ± 18 / 52 ± 17
Lhopitallier et al., [54]	France (60)	469 (65/53)	primary care	Medical patients with pneumonia	53 ± 18 / 50 ± 18
Mazlan et al., [34]	Malaysia (1)	85 (37/55)	ICU	Medical patients with VAP	49 ± 17 / 53 ± 17
Jeon et al., [35]	South-Korea (4)	52 (67/52)	ICU	Medical patients with sepsis	69 (61–75) / 70 (63–77)
Montassier et al., [56]	France (12)	285 (44/38)	ED	Medical patients with CAP	67 (46–83) / 67 (47–81)
O'Riordan et al., [55]	Ireland (1)	119 (39/58)	respiratory ward	Medical patients with LRTI	69 ± 14 / 68 ± 15
Daubin et al., [36]	France (11)	302 (29/34)	ICU	Medical patients with AECOPD	67 (61–76) / 67 (61–75)
Kip et al.,^c^ [37]	Netherlands (15)	1546 (39/40)	ICU	Medical patients with sepsis	65 (54–75) / 65 (57–75)
van der Does et al., [58]	Netherlands (2)	551 (48/44)	ED	Medical patients with fever	61 (43–70) / 62 (44–73)
Huang et al., [57]	USA (14)	1656 (57/57)	ED	Medical patients with LRTI	53 ± 18 / 53 ± 19
Liu et al., [38]	China (1)	98 (45/41)	ICU	Patients with sepsis	66 ± 9 / 65 ± 10
Xu et al., [16]	China (1)	156 (43/43)	ICU	Patients with sepsis	67 ± 9 / 65 ± 9
Slieker et al., [60]	Switzerland (1)	162 (42/43)	Surgical ward /ICU (88/12%)	Surgical patients with peritonitis	56 [36-73] / 57 [36-71]
Mahmutaj et al., [59]	Kosovo (1)	100 (32/38)	surgical ward	Surgical patients with acute abdomen	39 ± 20 / 47 ± 19
Ulm et al., [61]	Germany (10)	227 (54/57)	neurology ward	Medical patients after ACM stroke	76 ± 12 / 76 ± 11
Corti et al., [62]	Denmark (1)	120 (66/55)	ED	Medical patients with AECOPD	72 (64–80) / 73 (61–78)
Bloos et al., [39]	Germany (33)	1089 (NA)	ICU	Medical/surgical (43/57%) patients with sepsis	NA
de Jong et al., [40]	Netherlands (15)	1546 (39/40)	ICU	Medical patients with sepsis	65 (54–75) / 65 (57–75)
Lima et al., [63]	Brazil (1)	61 (44/52)	Hematology ward	Medical patients with febrile neutropenia (FN)	36 (26–53.8) / 33 (26.50)
Branche et al., 2015 [64]	USA (1)	150 (58/54)	General ward	Medical patients with non-pneumonic LRTI	61 (51–72) / 64 (50–74)
Drozdov et al., [65]	Switzerland (1)	125 (70/81)	ED	Medical patients with non-catheter-related UTI	71 (44–81) / 75 (51–80)
Verduri et al., [66]	Italy (18)	178 (13/14)	Respiratory ward	Medical patients with AECOPD	74 (69–78) / 73 (65–78)
Najafi et al., [41]	Iran (1)	60 (33/40)	ICU	Medical/surgical (88/12%) patients with SIRS	40 ± 18 / 41 ± 21
Ogasawara et al., [67]	Japan (1)	96 (55/46)	Respiratory ward	Medical patients with aspiration pneumonia	85 (81–92) / 87 (85–89)
Shehabi et al., [42]	Australia (11)	394 (53/40)	ICU	Medical surgical patients (88/12%) with suspected bacterial infection	63 ± 15 / 66 ± 16
Oliveira et al., [43]	Brazil (2)	94 (49/42)	ICU	Medical/surgical patients (86/14%) with severe sepsis or septic shock	60 ± 13 / 60 ± 19
Annane et al., [44]	France (8)	58 (20/32)	ICU	Medical/surgical patients (97/3%) with suspected sepsis	59 (40–67) / 54 (46–73)
Deliberato et al., [45]	Brazil (1)	81 (43/46)	ICU	Patients with sepsis	68 ± 21 / 62 ± 19
Tang et al., [68]	China (1)	260 (50/54)	ED	Medical patients with acute exacerbation of asthma	54 ± 14 / 55 ± 15
Layios et al., [46]	Belgium (1)	509 (48/39)	ICU	Medical/surgical patients (60/40%) with suspected sepsis	66 (55–76) / 65 (53–75)
Qu et al., [47]	China (1)	71 (29/28)	ICU	Medical patients with acute pancreatitis	43 ± 11 / 44 ± 11
Jensen et al., [48]	Denmark (9)	1200 (65/64)	ICU	Medical/surgical patients (59/41%) with sepsis	67 (58–76) / 67 (58–75)
Long et al., [69]	Japan (1)	162 (40/38)	ED	Medical patients with CAP	44 ± 16 / 47 ± 19
Maravić-Stojković et al., [49]	Serbia (1)	205 (30/33)	ICU	Patients after open heart cardiac surgery	60 ± 9 / 60 ± 10
Burkhardt et al., [70]	Germany (15)	550 (60/59)	Primary care	Medical patients with respiratory tract infection	41 ± 15 / 43 ± 16
Bouadma et al., [10]	France (7)	621 (33/35)	ICU	Medical/surgical patients (89/11%) with suspected bacterial infection	61 ± 15.2 / 62 ± 15
Stolz et al., [50]	Switzerland, USA (7)	101 (25/26)	ICU	Medical/surgical (52.5/47.5%) patients with VAP	53 [21–88] / 59 (18–83)
Kristoffersen et al., [71]	Denmark (3)	210 (48/46)	General ward	Medical patients with suspected LRTI	67 ± 18 / 67 ± 16
Hochreiter et al., [51]	Germany (1)	110 (49/ 46)	ICU	Surgical patients with sepsis	67 ± 14 / 67 ± 16
Schuetz et al., [72]	Switzerland (6)	1359 (40/45)	ED	Medical patients with LRTI	73 (59–82) / 72 (59–82)
Briel et al., [73]	Switzerland (53)	458 (58/62)	Primary care	Medical patients with respiratory tract infection	48 ± 18 / 48 ± 18
Schroeder et al., [52]	Germany (1)	27 (43/46)	ICU	Surgical patients with severe sepsis	69 ± 11 / 68 ± 14
Nobre et al., [53]	Switzerland (1)	68 (32/32)	ICU	Medical/surgical (75/25%) patients with sepsis	64 ± 12 / 70 ± 14
Stolz et al., [74]	Switzerland (1)	208 (51/59)	ED	Medical patients with AECOPD	70 (65–77) / 70 (65–79)
Christ-Crain et al., [75]	Switzerland (1)	302 (38/38)	ED	Medical patients with CAP	70 ± 17 / 70 ± 17
Christ-Crain et al., [76]	Switzerland (1)	243 (46/49)	ED	Medical patients with LRTI	63 ± 20 / 65 ± 17

^a^ presented as mean ± SD, median (IQR), median [range], ^b^ patients treated on wards under advanced supportive care because shortage of ICU beds, ^c^ cost-effectiveness analysis of de Jong et al., 2016; abbreviations: AECOPD—acute exacerbation of chronic obstructive pulmonary disease, CAP—community acquired pneumonia, ED—emergency department, ICU—intensive care unit, LRTI—lower respiratory tract infection, NA—not available, UTI—urinary tract infection, VAP—ventilator-associated pneumonia

We included mainly open-label, parallel group trials. One study had a factorial design [39]. Twenty studies recruited patients with suspected or confirmed infection/sepsis [10, 16, 30–32, 35, 37–48, 51–53], two studies included patients with ventilator-associated pneumonia (VAP) [34, 50]. We also included studies on acute exacerbation of COPD [36], aspiration pneumonia [33], pancreatitis [47] and one study on postoperative (cardiac surgery) patients [49]. PCT protocol was used to stop ABs [16, 30, 31, 34, 35, 37–40, 42, 43, 45, 50–53], to start ABs [41, 46, 49] or both [10, 32, 33, 36, 44, 47, 48]. Two studies [32, 43] used a predefined C-reactive protein (CRP) protocol in the control arm, all others used current AB guidelines.

## Primary outcome: length of antibiotic therapy

A meta-analysis of 21 RCTs [10, 16, 30, 31, 33–36, 38–40, 42–45, 47, 48, 50–53] with a total of 6669 patients revealed that the duration of AB therapy was reduced in the PCT-guided group compared to the SOC group (MD − 1.79 days, 95% CI: − 2.65, − 0.92, *p* < 0.001) (see Additional file 1: Figure S1).

**Fig. 2:**
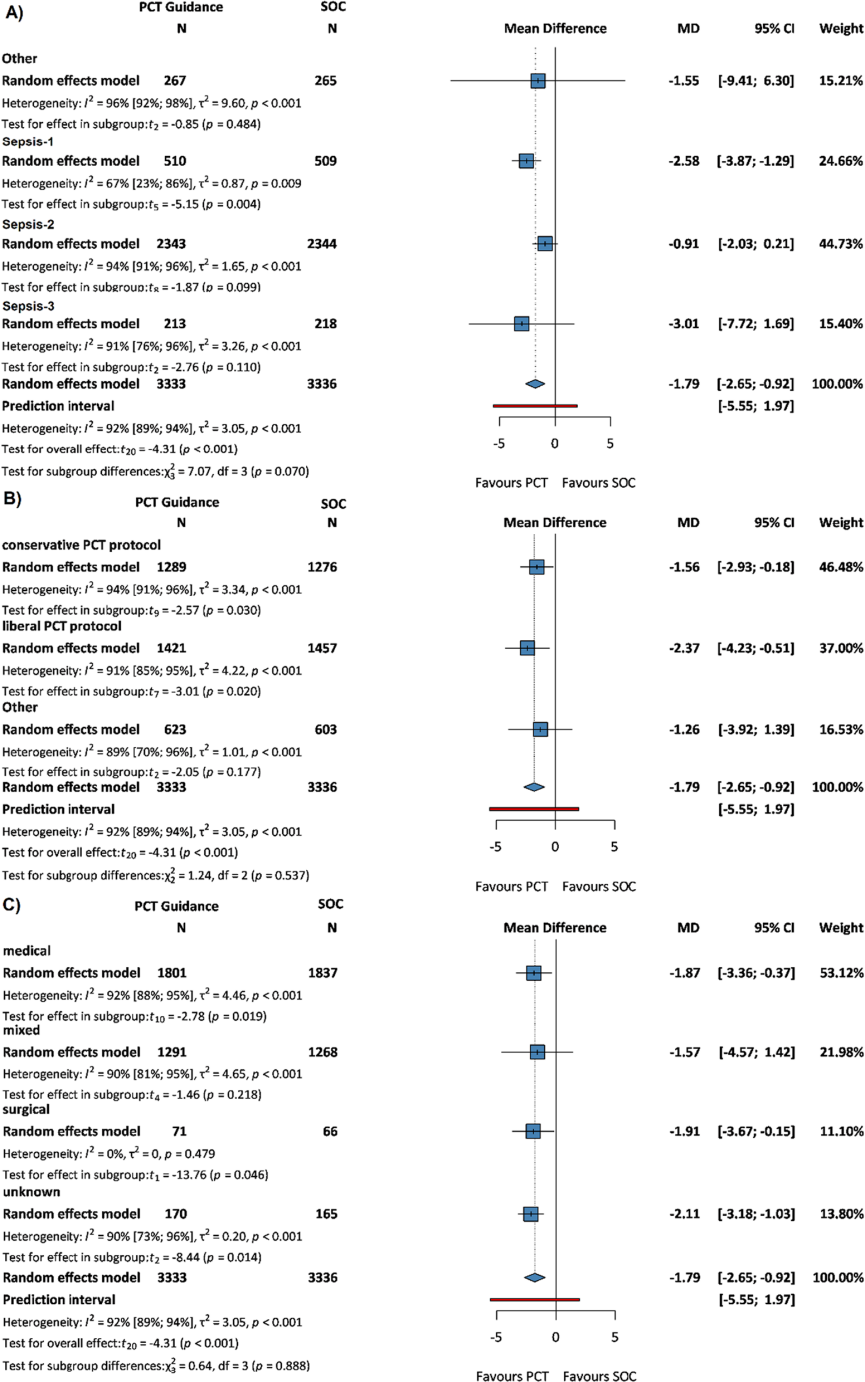
Forest plots representing the mean difference in length of AB therapy in **A** sepsis subgroups, **B** PCT protocol subgroups, and **C** patient population subgroups

This significantly reduced AB length was observed in Sepsis-1 patients (MD − 2.58 days, 95% CI: − 3.87, − 1.29, *p* = 0.004) (Fig. 2A), whether a conservative or liberal PCT protocol was used (MD − 1.56 days, 95% CI: − 2.93, − 0.18, *p* = 0.03 vs. − 2.37 days, 95% CI: − 4.23, − 0.51, *p* = 0.02 (Fig. 2B) and in the 100% medical patient population (MD − 1.87 days, 95% CI: − 3.36, − 0.37, *p* = 0.019) (Fig. 2C). In the Sepsis-3 cohort, the difference was non-significant (− 3.01 days, 95% CI − 7.72, 1.69).

## 28-day, ICU and in-hospital mortality

The odds of 28-day mortality and in-hospital mortality was reduced in PCT guidance compared to SOC, the former being statistically significant (OR 0.84, 95% CI: 0.74, 0.95, *p* = 0.008 (Additional file 1: Figure S2) and OR 0.85, 95% CI: 0.66, 1.10 (Additional file 1: Figure S3), respectively). There was no difference in ICU mortality between the two groups (OR 1.00, 95% CI: 0.74, 1.36) (Additional file 1: Figure S4).

**Fig. 3:**
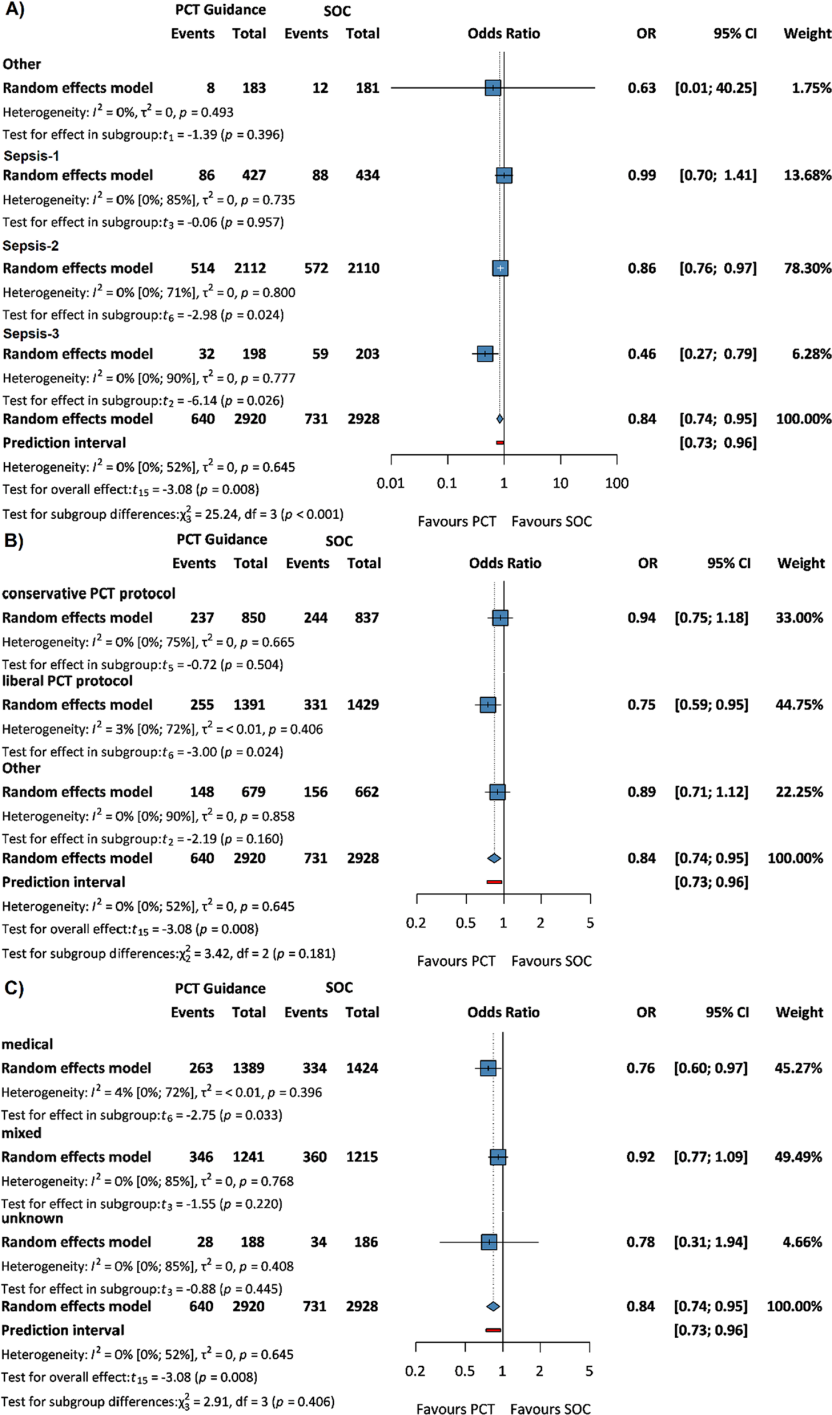
Forest plots representing the odds of 28-day mortality in **A** sepsis subgroups, **B** PCT protocol subgroups, and **C** patient population subgroups

This significantly reduced 28-day mortality was observed in Sepsis-2 and Sepsis-3 patients (OR 0.86, 95% CI: 0.76, 0.97, *p* = 0.024 and OR 0.46, 95% CI: 0.27, 0.79, *p* = 0.026, respectively) (Fig. 3A), applying liberal PCT protocol (OR 0.75, 95% CI: 0.59, 0.95, *p* = 0.024) (Fig. 3B) and in medical patients (OR 0.76, 95% CI: 0.60, 0.97, *p* = 0.033) (Fig. 3C).

## Recurrent and secondary infection

**Fig. 4:**
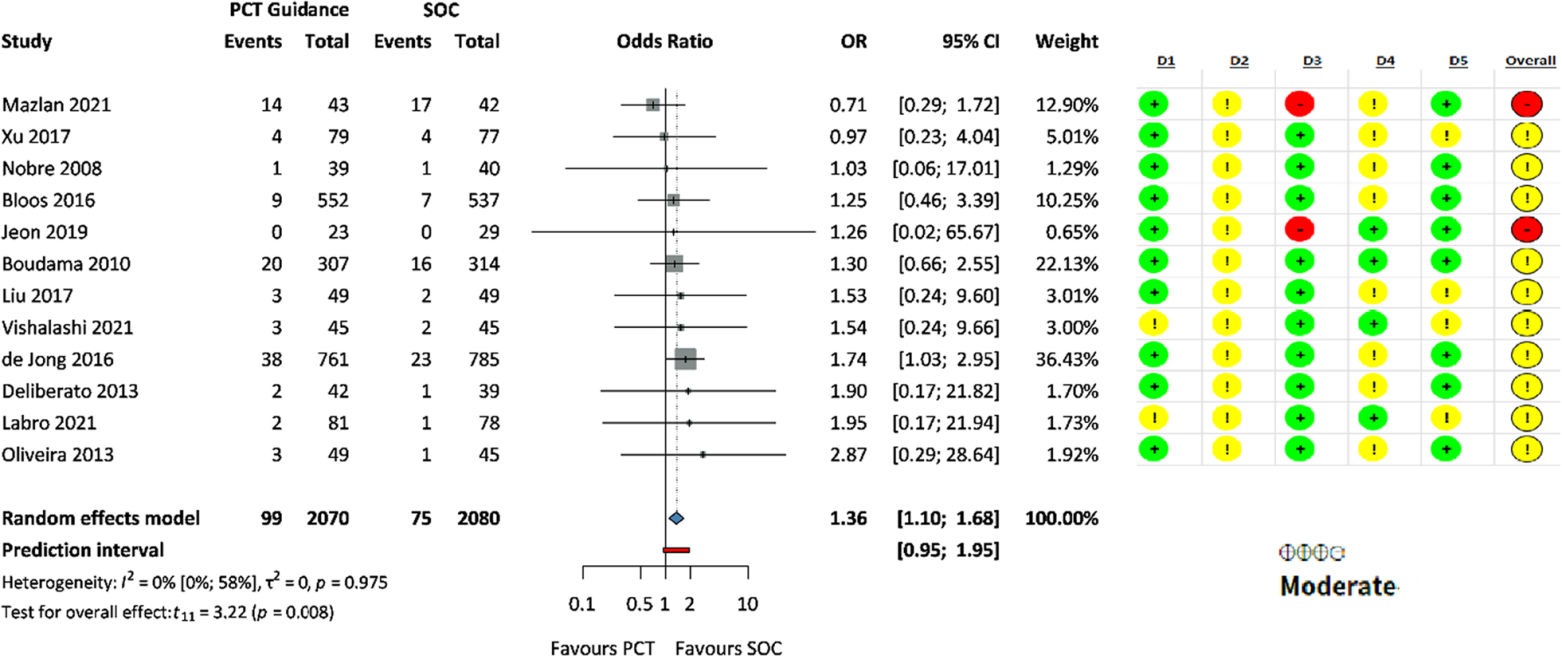
Forest plot representing the odds of recurrent infection

Infection recurrence was observed in 99 out of 2,070 patients in the PCT group and in 75 out of 2,080 patients in the SOC group, indicating a significant difference (OR 1.36, 95% CI: 1.10, 1.68, *p* = 0.008) (Fig. 4).

**Fig. 5:**
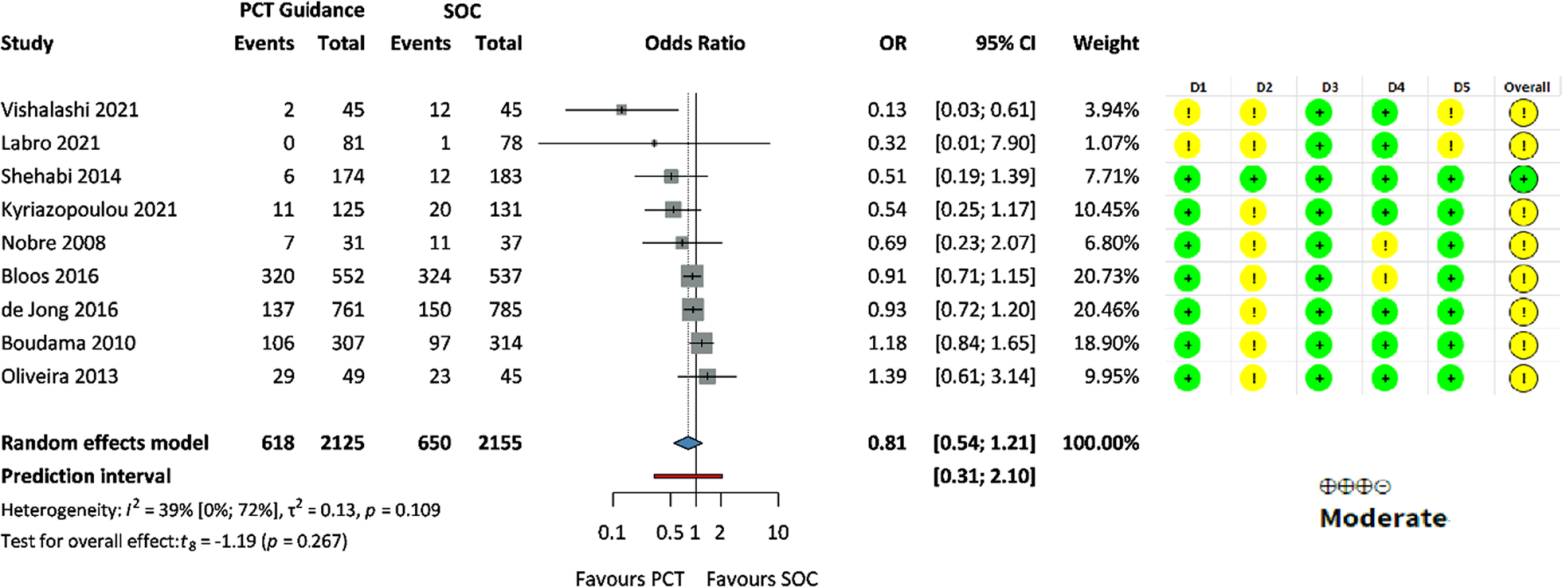
Forest plot representing the odds of secondary infection

There was no significant difference in the rate of secondary infections (OR 0.81, 95% CI: 0.54, 1.21) (Fig. 5).

## Length of ICU stay, length of hospital stay and healthcare costs

Length of ICU stay and length of hospital stay were non-significantly reduced in the PCT group compared to the SOC group (MD − 0.67 days 95% CI: − 1.76, 0.41 and MD − 1.23 days, 95% CI: − 3.13, 0.67, respectively) (Additional file 1: Figures S5 and S6). Due to the highly heterogeneous reporting of healthcare costs, we used a non-comprehensive method in the analysis, with results favoring PCT use (Additional file 1: Figure S7).

## Risk of bias and GRADE assessment

Two trials had high overall ROB due to missing outcome data [34, 35], whereas 23 trials had some concerns about ROB assessment due to deviations from intended intervention (PCT protocol violations) or the lack of reporting it [10, 16, 30–33, 36–41, 43–53]. Only one trial had overall low ROB [42]. For assessing publication bias, funnel plots can be found in the supplementary material (Additional file 1: Figure S8 (a-f)).

Certainty of evidence proved to be high for length of AB therapy and 28-day mortality. Moderate results were observed for in-hospital mortality, ICU mortality, rate of recurrent infection and rate of secondary infection, while GRADE was low for length of ICU stay and length of hospital stay and very low for healthcare costs. ROB and GRADE results are shown in the respective forest plots.

## Discussion

In our meta-analysis, we analyzed 26 RCTs [10, 16, 30–53] with a total of 9,048 patients, comparing the effects of PCT-guided AB therapy with standard of care on length of AB therapy, mortality, rate of recurrent and secondary infections, length of hospital and ICU stay and healthcare costs.

## Length of AB therapy

Our study confirms the findings of previous meta-analyses [77, 78] that PCT-guided AB therapy, including AB cessation rules can significantly reduce the length of AB therapy in ICU patients. An interesting finding in our study was that the three different sepsis definitions had an impact on the results, with significantly shorter AB therapy in the PCT group in the Sepsis-1 cohort and non-significant results in Sepsis-2 and 3 cohorts. Although the mean difference was by far the largest in Sepsis-3 patients [30, 31, 34], the results lacked statistical significance. The relatively low sample size of Sepsis-3 patients compared to other sepsis cohorts could be an explanation for the lack of significant results. On the other hand, five out of nine trials in the Sepsis-2 cohort used conservative PCT protocols, two of them [43, 48] demonstrated even longer AB duration in the PCT group, which may also have contributed to the observed smaller effect on AB length in this patient population.

We further classified the trials into two subgroups (liberal and conservative) depending on the stopping rule in the PCT group except for three trials [39, 51, 52] that used a very unique protocol and studies using only starting rules that did not report this outcome [41, 46, 49]. Our analysis overtly suggests that a liberal PCT protocol may result in shorter AB duration compared with a conservative one. Furthermore, protocol adherence was very low (40–50%) in three trials of the group using the liberal protocol [10, 35, 40], so the difference could have been larger with fewer protocol violations.

Our results show that in mixed populations (the proportion of surgical patients is at least 25%), the length of AB therapy is slightly longer than in medical patients. Apart from one study with different PCT cut-offs for patients during the 48-h postoperative period [44], the trials included used the same protocol regardless of the population. PCT values can be elevated after surgery even in the absence of infection [79], and the use of absolute PCT stopping thresholds in these cases might result in AB overuse. Data on populations including only surgical patients were insufficient for meta-analysis, but pooling data from two surgical cohorts [51, 52] results in an even more pronounced reduction in the length of AB therapy. This may be explained by the high absolute stopping threshold (1 ng/mL) used in the study protocols.

## 28-day, in-hospital and ICU mortality

Our results suggest that 28-day and in-hospital mortality is lower in the PCT group than in the SOC group. However, results are conflicting, as some trials showed survival benefit [30, 40], and some others did not [10, 43, 48]. This contradiction may be partially resolved by our results, namely that mortality benefit is only observed in Sepsis-2 and Sepsis-3 patients, medical patients and trials using liberal PCT protocol, all of which are associated with shorter AB duration. Unfortunately, our results do not allow us to explain the relationship between AB therapy duration and mortality. Nevertheless, several studies have shown the potential harmful effects of ABs. These include direct toxic effects and organ injury [80], development of AB resistance and potentially higher chances of secondary infections, mostly caused by MDRO [1], mitochondrial dysfunction associated with ABs [81] and injury and collapse of the microbiome [82]. Moreover, an initial low PCT value can help the differential diagnosis, thereby optimizing patient care and reducing mortality.

## Recurrent and secondary infections

Theoretically, too short course of ABs could risk infection recurrence, while overuse of ABs is a risk for secondary infections. Our data show significantly higher recurrence of infection in the PCT group, which contradicts the latest meta-analysis [77]; however, they included mostly non-ICU patients with respiratory tract infections. We share the view of the open-label SAPS trial group [40] that bias cannot be excluded, as clinicians might think of a reinfection sooner in the PCT group. The results on secondary infections are conflicting; hence, PCT guidance had uncertain effects on this outcome.

## Length of ICU stay, length of hospital stay, healthcare costs

Higher infection recurrence rate did not result in excessive ICU and in-hospital stay in the PCT group, which is consistent with the previous meta-analysis in septic ICU patients [78]. Despite the high heterogeneity in cost-effectiveness reports, our results suggest that PCT guidance at least does not appear to be inferior to SOC, but further research is needed to draw firm conclusions about this outcome.

## PCT-guided AB therapy outside the ICU

We included 23 RCTs [54–76] in our review. Eighteen studies recruited patients with respiratory tract infections treated in ED [56, 57, 62, 64, 68, 69, 72, 74–76], general ward [55, 61, 66, 67, 71] or primary care [54, 70, 73]. Two trials included patients with peritonitis [59, 60] and one study each included patients with fever [58], febrile neutropenia [63] and urinary tract infection (UTI) [65]. Some studies used additional diagnostics: thoracic ultrasound [54] or viral PCR [64]. In studies on respiratory tract infection, AB use was either reduced in the PCT group or similar between study arms with no difference in adverse outcomes. In patients with peritonitis, Mahmutaj et al. reported a significant reduction in AB use in the PCT arm without an elevated risk of infection recurrence [59]. Slieker et al. in a similar trial reported no adverse outcomes associated with non-significantly reduced AB treatment duration [60]. An approach based on PCT and pyuria in UTI patients [65] reduced AB exposure by 30% without adverse effects, whereas in febrile neutropenia, [63] PCT had no effect on AB use.

## Strengths and limitations

To the best of our knowledge, this meta-analysis contains the largest number of studies to date, all of which are RCTs. We are also the first to perform subgroup analyses based on sepsis definitions, patient populations, and PCT protocols: our results provide some support that recruiting patients into studies according to the Sepsis-3 definition may have an impact on outcomes; that surgical and medical patients may require separate treatment protocols; and conservative guidance is not superior to a liberal strategy. Finally, we rigorously followed all Cochrane Collaboration guidelines, thereby ensuring maximum quality, transparency, and reproducibility of the results.

Our meta-analysis has certain limitations. First, in the control arm, SOC was not “standardized” as different AB guidelines were applied in different institutions that could potentially result in longer duration of AB therapy in some regions, thus overestimating the effect of PCT guidance. Second, “PCT guidance” does not mean a standard approach, as studies applied different PCT protocols: 16 out of 26 included studies used PCT protocol to stop ABs, 3 used PCT protocol to start ABs, while 7 used PCT guidance for both starting and stopping AB therapy. Furthermore, not all studies reported on all outcomes. The source of infection varied between the studies and the number of patients with septic shock ranged between 7 and 87%, indicating a huge variability in severity of patient populations on the one hand and, on the other hand, a possible impact on outcomes cannot be excluded according to the 15 studies reporting PCT protocol adherence, which ranged between 44 and 97%. Furthermore, AB appropriateness could have an important effect on outcome. However, we do not know whether patients received appropriate or inappropriate ABs in the same or similar proportion in the PCT-guided and control groups as this outcome was only reported in 5 studies in which the groups were well balanced in this regard [10, 30, 35, 45, 50], but we still cannot draw conclusions on this topic. Finally, almost all studies excluded immunocompromised patients in their medical history; therefore, the generalizability of our results is limited.

## Implications for practice and research

The rapid application of scientific results is of utmost importance [83, 84]. Our results suggest that PCT-guided AB management could reduce the length of AB therapy in ICU patients, especially in countries and institutes where routine AB administration exceeds 7 days.

The current sepsis guideline [5] recommends against the use of PCT and clinical evaluation to decide when to start AB therapy in septic patients. However, we believe that further research is needed in this field, especially to evaluate PCT kinetics (i.e., changes in 12–24 h) compared to protocols based on a fix value (i.e., 0.5 ng/mL as cut off) [79, 85]. Furthermore, the increased rate of recurrent infections, the difference between medical and surgical patients and finally testing whether a liberal or a conservative regime is more beneficial should also deserve further investigations. We also suggest that in future trials, “organ support free days” should be used as the primary outcome [86] rather than mortality, which is affected by a number of confounding factors during the full course of a critical illness; therefore it may not necessarily reflect the efficacy of a particular intervention. Finally, we need data on immunocompromised patients who may also benefit from this approach.

## Conclusion

PCT-guided AB therapy may be associated with reduced AB use, lower 28-day mortality but higher infection recurrence, with similar ICU and hospital length of stay. Our results render the need for better designed studies investigating the role of PCT-guided AB stewardship in critically ill patients.

### Supplementary Information

**Additional file1**: Table S1 Prisma Checklist 2020. Table S2 Other characteristics of included studies. Figure S1 Length of AB therapy. Figure S2 28-day mortality. Figure S3 In-hospital mortality. Figure S4 ICU mortality. Figure S5 Length of ICU stay. Figure S6 Length of hospital stay. Figure S7 Healthcare costs Figure S8 (a-f) Funnel plots

## Data Availability

The datasets used in this study can be found in the full-text articles included in the systematic review and meta-analysis.

## Abbreviations

AB: Antibiotic
ABs: Antibiotics
AECOPD: Acute exacerbation of chronic obstructive pulmonary disease
CAP: Community acquired pneumonia
CI: Confidence interval
COPD: Chronic obstructive pulmonary disease
CRP: C-reactive protein
ED: Emergency department
GRADE: Grading of Recommendations Assessment, Development, and Evaluation
ICU: Intensive care unit
LOS: Length of stay
LRTI: Lower respiratory tract infection
MD: Mean difference
MDRO: Multidrug-resistant organism
OR: Odds ratio
PCR: Polymerase chain reaction
PCT: Procalcitonin
RCT: Randomized controlled trial
RCTs: Randomized controlled trials
ROB: Risk of bias
SD: Standard deviation
SE: Standard error
SOC: Standard of care
SOFA: Sequential organ failure assessment or Sepsis-related organ failure assessment
UTI: Urinary tract infection
VAP: Ventilator-associated pneumonia
