# Supplementary material for: Procalcitonin-guided antibiotic therapy may shorten length of treatment and may improve survival—a systematic review and meta-analysis
Source: Crit Care. 2023 Oct 13;27:394. doi: 10.1186/s13054-023-04677-2 (PMC10576288; doi:10.1186/s13054-023-04677-2)

Supplementary material

Contents

[Table S1: PRISMA checklist 1](#_Toc145618762)

[Table S2: Other characteristics of included studies 4](#_Toc145618763)

[Figure S1: Forest plot of length of AB therapy 19](#_Toc145618764)

[Figure S2: Forest plot of 28-day mortality 20](#_Toc145618765)

[Figure S3: Forest plot of in-hospital mortality 21](#_Toc145618766)

[Figure S4: Forest plot of ICU mortality 22](#_Toc145618767)

[Figure S5: Forest plot of length of ICU stay 23](#_Toc145618768)

[Figure S6: Forest plot of length of hospital stay 24](#_Toc145618769)

[Figure S7: Forest plot of healthcare costs 25](#_Toc145618770)

[Figure S8: Funnel plots 25](#_Toc145618771)

# Table S1: PRISMA checklist

| **Section and Topic** | **Item #** | **Checklist item** | **Location where item is reported** |
| --- | --- | --- | --- |
| **TITLE** | | |  |
| Title | 1 | Identify the report as a systematic review. | 1 |
| **ABSTRACT** | | |  |
| Abstract | 2 | See the PRISMA 2020 for Abstracts checklist. | 3-4 |
| **INTRODUCTION** | | |  |
| Rationale | 3 | Describe the rationale for the review in the context of existing knowledge. | 5 |
| Objectives | 4 | Provide an explicit statement of the objective(s) or question(s) the review addresses. | 6 |
| **METHODS** | | |  |
| Eligibility criteria | 5 | Specify the inclusion and exclusion criteria for the review and how studies were grouped for the syntheses. | 6-7 |
| Information sources | 6 | Specify all databases, registers, websites, organisations, reference lists and other sources searched or consulted to identify studies. Specify the date when each source was last searched or consulted. | 7 |
| Search strategy | 7 | Present the full search strategies for all databases, registers and websites, including any filters and limits used. | 7 |
| Selection process | 8 | Specify the methods used to decide whether a study met the inclusion criteria of the review, including how many reviewers screened each record and each report retrieved, whether they worked independently, and if applicable, details of automation tools used in the process. | 7 |
| Data collection process | 9 | Specify the methods used to collect data from reports, including how many reviewers collected data from each report, whether they worked independently, any processes for obtaining or confirming data from study investigators, and if applicable, details of automation tools used in the process. | 7 |
| Data items | 10a | List and define all outcomes for which data were sought. Specify whether all results that were compatible with each outcome domain in each study were sought (e.g. for all measures, time points, analyses), and if not, the methods used to decide which results to collect. | 7 |
|  | 10b | List and define all other variables for which data were sought (e.g. participant and intervention characteristics, funding sources). Describe any assumptions made about any missing or unclear information. | 7 |
| Study risk of bias assessment | 11 | Specify the methods used to assess risk of bias in the included studies, including details of the tool(s) used, how many reviewers assessed each study and whether they worked independently, and if applicable, details of automation tools used in the process. | 8 |
| Effect measures | 12 | Specify for each outcome the effect measure(s) (e.g. risk ratio, mean difference) used in the synthesis or presentation of results. | 8 |
| Synthesis methods | 13a | Describe the processes used to decide which studies were eligible for each synthesis (e.g. tabulating the study intervention characteristics and comparing against the planned groups for each synthesis (item #5)). | 8 |
|  | 13b | Describe any methods required to prepare the data for presentation or synthesis, such as handling of missing summary statistics, or data conversions. | 8 |
|  | 13c | Describe any methods used to tabulate or visually display results of individual studies and syntheses. | 8-9 |
|  | 13d | Describe any methods used to synthesize results and provide a rationale for the choice(s). If meta-analysis was performed, describe the model(s), method(s) to identify the presence and extent of statistical heterogeneity, and software package(s) used. | 8-9 |
|  | 13e | Describe any methods used to explore possible causes of heterogeneity among study results (e.g. subgroup analysis, meta-regression). | 7 |
|  | 13f | Describe any sensitivity analyses conducted to assess robustness of the synthesized results. | 8-9 |
| Reporting bias assessment | 14 | Describe any methods used to assess risk of bias due to missing results in a synthesis (arising from reporting biases). | 8 |
| Certainty assessment | 15 | Describe any methods used to assess certainty (or confidence) in the body of evidence for an outcome. | 8 |
| **RESULTS** | | |  |
| Study selection | 16a | Describe the results of the search and selection process, from the number of records identified in the search to the number of studies included in the review, ideally using a flow diagram. | 10 |
|  | 16b | Cite studies that might appear to meet the inclusion criteria, but which were excluded, and explain why they were excluded. | 10 |
| Study characteristics | 17 | Cite each included study and present its characteristics. | 10 |
| Risk of bias in studies | 18 | Present assessments of risk of bias for each included study. | forest plots |
| Results of individual studies | 19 | For all outcomes, present, for each study: (a) summary statistics for each group (where appropriate) and (b) an effect estimate and its precision (e.g. confidence/credible interval), ideally using structured tables or plots. | forest plots |
| Results of syntheses | 20a | For each synthesis, briefly summarise the characteristics and risk of bias among contributing studies. | forest plots |
|  | 20b | Present results of all statistical syntheses conducted. If meta-analysis was done, present for each the summary estimate and its precision (e.g. confidence/credible interval) and measures of statistical heterogeneity. If comparing groups, describe the direction of the effect. | forest plots |
|  | 20c | Present results of all investigations of possible causes of heterogeneity among study results. | forest plots |
|  | 20d | Present results of all sensitivity analyses conducted to assess the robustness of the synthesized results. | forest plots |
| Reporting biases | 21 | Present assessments of risk of bias due to missing results (arising from reporting biases) for each synthesis assessed. | forest plots |
| Certainty of evidence | 22 | Present assessments of certainty (or confidence) in the body of evidence for each outcome assessed. | forest plots |
| **DISCUSSION** | | |  |
| Discussion | 23a | Provide a general interpretation of the results in the context of other evidence. | 16-19 |
|  | 23b | Discuss any limitations of the evidence included in the review. | 20 |
|  | 23c | Discuss any limitations of the review processes used. | 20 |
|  | 23d | Discuss implications of the results for practice, policy, and future research. | 20-21 |
| **OTHER INFORMATION** | | |  |
| Registration and protocol | 24a | Provide registration information for the review, including register name and registration number, or state that the review was not registered. | 6 |
|  | 24b | Indicate where the review protocol can be accessed, or state that a protocol was not prepared. | 6 |
|  | 24c | Describe and explain any amendments to information provided at registration or in the protocol. | 6 |
| Support | 25 | Describe sources of financial or non-financial support for the review, and the role of the funders or sponsors in the review. | 2 |
| Competing interests | 26 | Declare any competing interests of review authors. | 2 |
| Availability of data, code and other materials | 27 | Report which of the following are publicly available and where they can be found: template data collection forms; data extracted from included studies; data used for all analyses; analytic code; any other materials used in the review. | 3 |

# Table S2: Other characteristics of included studies

| **Study author, year**  **(follow up time)** | **Exclusion criteria** | **PCT protocol** | | | **Appropriateness of AB therapy** | **days of AB therapy ^a^ (PCT vs control)** | **safety outcome (PCT vs control)** |
| --- | --- | --- | --- | --- | --- | --- | --- |
|  |  | **start AB if** | **stop AB if** | **Adherence** |  |  |  |
| Kyriazopoulou et al.,2021  (180 days) | - need for prolonged treatment - viral or parasite infections - tuberculosis, cystic fibrosis - neutropenia, infection by HIV with low CD4 count - pregnancy or lactation | - none | - PCT reduced > 80% of baseline value or - PCT < 0,5 ng/mL on day 5 or later | 77% | 98.0%/98.2% (PCT/control arm) | 5 (5-7) vs 10 (7-15) | 28-day mortality: 15.2% vs 28.0% |
| Ali et al., 2021  (NA) | - first doses of AB received > 24 h before screening - pregnancy - immunocompromised patients - chronic kidney disease/chronic liver failure - bacterial endocarditis | - PCT ≥ 0.25 ng/mL | - PCT reduced > 80-90% of initial value or - PCT < 0.5 ng/mL and no clinical signs of infection | NA | NA | NA | 28-day mortality: 26.7% vs 50.0% |
| Vishalashi et al., 2021  (until discharge) | - pregnancy - need for prolonged AB therapy (e.g. infective endocarditis) - viral or parasitic infections - severe immunosuppression - admission for postoperative observation - estimated LOS in ICU < 24 hours | - none | - PCT reduced > 80% of baseline value or - PCT < 0.1 ng/mL | NA | NA | 5.0±2.6 vs 7.7±3.0 | ICU mortality: 8.9% vs 15.6% |
| Labro et al., 2021  (90 days) | - pregnancy - inclusion in another interventional clinical study - moribund patients - trauma patients, surgery within the last 4 days, cardiorespiratory arrest, administration of anti-thymocyte globulin - immunosuppression - absolute indication for administration of ABs at the time of ICU admission (meningitis, pneumonia) - chronic infection with prolonged AB treatment - patients with hemodynamic instability of septic origin or respiratory insufficiency (PaO_2_/FiO_2_ ratio ≤ 200 mmHg and PEEP ≥ 5 cmH_2_O). | - PCT > 0.5 ng/mL in the first 3 days after randomization | - PCT reduced > 80% of baseline value or - PCT < 0.5 ng/mL | 84% (for starting AB) | NA | 3.0±4.1 vs 2.3±3.4 | 28-day mortality: 6.2% vs 11.5% |
| Lhopitallier et al., 2021  (28 days) | - previous AB therapy for the current episode - acute sinusitis or non infective disorder - previous episode of AECOPD treated with AB during the last 6 months - pregnancy - severe immunodeficiency - decision by the general practitioner (GP) to admit the patient - GP not available for performing study - lack of informed consent | - PCT > 0.25 ng/mL | - none | NA | NA | patients on ABs by day 28: 40% vs 70% | serious adverse outcome by day 28: 10% vs 8% |
| Mazlan et al., 2021  (28 days) | - AB prophylaxis or AB treatment not for VAP - selective decontamination of the digestive tract - prolonged therapy (e.g. endocarditis) - expected ICU stay < 24 h - severe immunosuppression - severe infections due to non-bacterial causes - previously enrolled in the study | - none | - PCT reduced > 80% of peak value or - PCT < 0.5 ng/mL | 72.1 % | NA | 10.3±2.7 vs 11.5±3.1 | 28-day mortality: 11.6% vs 16.7% |
| Jeon et al., 2019  (NA) | - bacterial infection requiring more than 3 weeks of AB therapy - AB therapy started 48 hours or more before enrollment - severely immunocompromised patients, patients on immunosuppressive therapy - patients not expected to survive to hospital discharge - do-not-resuscitate orders - pregnancy | - none | - PCT reduced > 80% of peak value or - PCT< 0.5 ng/mL | 57% | 83%/76% (PCT/control arm) | 10 (8-14) vs 13 (10-21) | 28-day mortality: 17.4% vs 20.7% |
| Montassier et al., 2019  (30 days) | - pregnancy - immunosuppression - exacerbation of COPD or a life-threatening presentation expected to lead to possible imminent death - who had received antibiotics for the current episode of illness - who were anticipated to be discharged from the ED within 6 hours of their registration | - PCT > 0.5 ng/mL (strongly recommended) - or PCT > 0.25 ng/mL (recommended) | - PCT < 0.25 ng/mL | 75% | NA | 10 (7-14) vs 9 (6-13) | rate of adverse events: 15% vs 20% |
| O'Riordan et al., 2019  (30 days) | - unable to give written informed consent - cognitive impairment or severe dementia - readmission to hospital within 30 days of previous admission - immunosuppression - life-threatening medical comorbidities leading to possible imminent death - do not resuscitate (DNR) status - chronic infections necessitating prolonged AB treatment (cystic fibrosis, infective endocarditis etc.) - 24 h of appropriate AB therapy prior to initial PCT level - active IVDU (intravenous drug user) - pregnancy | - PCT > 0.5 ng/mL (strongly recommended) - or PCT > 0.25 ng/mL (recommended) | - PCT < 0.25 ng/mL (recommended) - PCT < 0.1 ng/mL (strongly recommended) | 35% | NA | 8.9±6.3 vs 11.0±7.6 | infection relapse: 7.6% vs 20.0% |
| Daubin et al., 2018  (3 months) | - pregnancy - clinical evidence of infection other than LRTI - severe acute asthma - moribund patients/suffering from a disease with an estimated survival time < 2 months - severe immunosuppression - nosocomial infection - known PCT level at the time of ICU admission | - 0.1< PCT < 0.25 ng/mL (encouraged) - PCT > 0.25 ng/mL (strongly encouraged*)* | - PCT reduced > 90% of peak value or - PCT < 0.1 ng/mL | NA | NA | 5.2±6.5 vs 5.4±4.4 | 28-day mortality: 12.6% vs 11.3% |
| Kip et al., 2018  (cost analysis of de Jong, 2016)  (1 year) | - AB prophylaxis - AB solely as part of selective decontamination of the digestive tract - prolonged therapy (e.g. endocarditis) - expected ICU stay of less than 24 h - severe immunosuppression - severe infections (due to viruses, parasites, Mycobacterium tuberculosis) - moribund patients | - none | - PCT reudced > 80% of peak value or - PCT < 0.5 ng/mL | 44% within 24 h and 97% within 48 h after reaching stopping value | NA | NA | NA |
| van der Does et al., 2018  (30 days) | - immunocompromised conditions - pregnancy - moribund patients - patients < 72 h after surgery or requiring primary surgical intervention | - PCT > 0.5 ng/mL | - none | 56% | NA | AB prescribed: 73% vs 77% | combined safety endpoint: 11% vs 16% |
| Huang et al., 2018  (30 days) | - physicians were unlikely to withhold ABs (prior ABs, vasopressor use, invasive mechanical ventilation, severe immunosuppression, accompanying non-respiratory infection, known lung abscess/empyema) - chronic dialysis, metastatic cancer, surgery in the past seven days - follow-up would be difficult (prisoners, homeless) | - PCT > 0.5 ng/mL (strongly recommended) - or PCT > 0.25 ng/mL (recommended) | - PCT < 0.25 ng/mL (recommended) - PCT < 0.1 ng/mL (strongly recommended) | 72.9% in ED, 64.8% in the entire period | NA | 4.2±5.8 vs 4.3±5.6 | adverse outcomes: 11.7% vs 13.1% |
| Liu et al., 2017  (30 days) | - AB profilaxis - patients who once took ABs due to virus infection and bacterial infection - malignant tumor - no estimated survival and leaving the hospital | - none | - PCT reduced > 90% of peak value or - PCT < 0.25 ng/mL | NA | NA | 7.7±0.6 vs 10.2±0.8 | 30-day mortality: 10.2% vs 12.2% |
| Xu et al., 2017  (28 days) | - surgical AB prophylaxis - bacterial infection requiring AB treatment (tuberculosis etc.) for more than 3 weeks - fungal/viral infection - immunosuppressant use - medullary thyroid carcinoma, small cell lung cancer - can not be expected to survive until discharge | - none | - PCT reduced > 90% of peak value or - PCT <0.25 ng/mL | NA | NA | 8.3±0.3 vs 10.1±0.4 | 28-day mortality: 12.3% vs 11.7% |
| Slieker et al., 2017  (30 days) | - any kind of immunosuppression - medullary cancer of the thyroid - severe hepatocellular insufficiency - spontaneous peritonitis in patients with ascites | - none | - PCT <1 ng/mL on day 3 or - PCT <0.25 ng/mL or < 20% of baseline value after day 3 | NA | NA | 8 (5-16) vs 10 (6-12) | 30-day mortality: 3,8% vs 9,4% |
| Mahmutaj et al., 2017  (NA) | - autoimmune disease - acute hepatic insufficiency - diabetes - immunosuppression - pregnancy - corticosteroid therapy - no informed consent | - none | - if PCT reduced > 80% of initial value or - PCT < 0.5 ng/mL | NA | NA | 10.6±6.6 vs 13.2±4.2 | infection recurrence: 4% vs 6 % |
| Ulm et al., 2017  (6 months) | - intracerebral hemorrhage or lacunar infarction - use of AB within the last 10 days - suspected life expectancy < 3 months - modified Rankin Scale (mRS) before stroke onset ≥4 - participation in other interventional trials - pregnancy/lactation. | - PCT > 0.05 ng/mL | - none | 65% | NA | 3 (0-6) vs 0 (0-5) | mortality: 4% vs 5% |
| Corti et al., 2016  (28 days) | - patient unable to understand or respond to oral or written information - previously been enrolled in the study - do not resuscitate order in place | - PCT > 0.25 ng/mL | - after 3,5,7 days if initial PCT < 0.5 ng/mL, 0.5 ng/mL < PCT < 1 ng/mL, and 1 ng/mL < respectively - PCT reduced > 80% of peak value (5-10 ng/mL initial value) | 61% | NA | 3.5 (0-10) vs 8.5 (1-11) | composite harm endpoint: 35% vs 26% |
| Bloos et al., 2016  (90 days) | - pregnant and lactating women - patients with selenium intoxication - individuals with infections for which guidelines recommend a longer duration of antimicrobial therapy - immunocompromised patients - those without commitment for full therapy or where death was imminent | - none | - PCT level ≤ 1 ng/mL (after day 4) or - PCT level dropped by at least 50% compared to previous value - PCT measurement on day 0, 1, 4, 7, 10, and 14 | 69% at day 4; 40.9% at day 7 | NA | 7 (3-12) vs 7 (3-12) | 28-day mortality: 25.6% vs 28.2% |
| de Jong et al., 2016  (1 year) | - AB prophylaxis - AB solely as part of selective decontamination of the digestive tract - prolonged therapy (eg, endocarditis) - expected ICU stay of less than 24 h - severe immunosuppression - severe infections (due to viruses, parasites, Mycobacterium tuberculosis) - moribund patients | - none | - PCT reduced > 80% of peak value or - PCT < 0.5 ng/mL | 44% within 24 h and 97% within 48 h after reaching stopping value | NA | 5 (3-9) vs 7 (4-11) | 28-day mortality: 19.6% vs 25.0% |
| Lima et al., 2016  (90 days) | - severe organ dysfunction (e.g. hypotension, ICU admission, DIC) - previous invasive fungal infection - infections due to Pseudomonas spp., Acinetobacter spp., Staphylococcus aureus, Mycobacterium tuberculosis,Pneumocystis jirovecii, Toxoplasma gondii or HIV - infections requiring AB for a long time (e.g. infective endocarditis, osteomyelitis) - grade 3 or 4 oral mucositis - pregnancy | - none | - PCT reduced > 90% of peak level or - PCT < 0.5 ng/mL for two days in a row regardless of initial values | 73% | 58.1% / 54.8% (PCT / control group) | 9 (5.8-12.0) vs 8 (4-11) | clinical cure: 60.0% vs 54.8% |
| Branche et al., 2015  (NA) | - Patients with high risk for bacterial infection (e.g. ICU requirement, active chemotherapy or radiation, immunosuppression) - definitive infiltrate on chest radiograph - enrollment systolic blood pressure of < 90 mmHg, and ≥15% band forms in peripheral blood | - 0.25 ng/mL < PCT < 0.49 ng/mL (encouraged) - PCT ≥ 0.5 ng/mL (strongly encouraged) - + use of viral PCR | - none | 64% | NA | 3 (1-7) vs 4 (0-8) | serious adverse events: 3% vs 3% |
| Drozdov et al., 2015  (90 days) | - other infection than UTI - AB therapy within 48 hours before presentation - pregnancy - prostatitis - foreign bodies within the urinary tract - endovascular prosthesis - non-endovascular prosthesis within 6 months after implantation - insufficient language skills with no possibility   for translation   - foreseeable non-compliance for follow-up - e.g. current drug abuse; severe immunodeficiency - hemodialysis, transplant patients, or life threatening medical comorbidities leading to possible imminent death | - none | - PCT reduced > 80% of peak value and pyuria normalized or - PCT < 0.25 ng/mL | NA | NA | 6 (4–8) vs 10 (7–11) | infection recurrence: 25% vs 22% |
| Verduri et al., 2015  (6 months) | - bronchial asthma - unstable concomitant disease (cardiovascular, renal, hepatic, gastrointestinal, neurological, metabolic, musculoskeletal, neoplastic, respiratory or other disease) - pregnancy/breastfeeding - clinically significant laboratory abnormalities suggestive of unstable concomitant disease - survival for 1 year unlikely - inability to give written consent | - minimum 3 days of AB | - measure on day 1, 2 and 3 - if a single PCT > 0.25 ng/mL, AB for 10 days - if one PCT is 0.1 ng/ml < and < 0.25 ng/mL, stop AB on day 3 or - stop AB if all PCT results are <0.1 ng/mL or there is a consistent trend towards PCT normalization with the latest value < 0.1 ng/mL | NA | NA | not reported | hospital (AECOPD) readmission: 15% vs 9% |
| Najafi et al., 2015  (NA) | - documented infection, pus from wound or abscess, empyema, thrombophlebitis - infection due to viruses or parasites - hypoxemia (PaO2 < 60 mmHg) - oliguria (urine output < 30 ml/hr) - Glasgow Coma Scale (GCS) 3 without sedation - parenteral AB usage 24 hours before admission to ICU - hospitalization 48 hours before enrollment - conditions requiring prolonged AB therapy (endocarditis, osteomyelitis) - severely immunocompromised patients | - PCT ≥ 2 ng/mL - if PCT < 0.5 ng/mL, recheck in 12h, - if PCT 0.5-2 ng/mL, recheck in 8 h | - none | NA | NA | total AB exposure days: 128 vs 320 days | in hospital mortality: 16.6 vs 13.3% |
| Ogasawara et al., 2014  (30 days) | - known severe allergy to any drugs - sepsis or a severe infectious disease - severe underlying disease (malignancy, COPD, heart failure) that affected the prognosis - patients who could not safely have cessation of oral intake or hydration as a treatment for aspiration pneumonia because of dementia | - none | - stop AB after 3, 5, or 7 days depending on initial PCT levels (< 0.5 ng/mL,0.5–1.0 ng/mL, or > 1.0 ng/mL respectively) or - if PCT reduced > 90 % of peak value (if initial value was > 5.0 ng/mL) | NA | NA | 5 (4-7) vs 8 (6-10) | relapse and 30 day mortality: 25.0% vs 37.5% |
| Shehabi et al., 2014  (90 days) | - AB for surgical prophylaxis - proven bacterial infection requiring more than 3 weeks of AB therapy - isolated systemic fungal or systemic viral infection - neutropenia with a neutrophil count less than 1000 cells/mm^3^ receiving immunosuppressive agents - cardiac surgery or trauma or heat stroke within 48 hours - medullary thyroid carcinoma or small cell lung cancer - not expected to survive to hospital discharge - pregnancy | - none | - if PCT < 0.1 ng/mL or - if 0.1 ng/mL < PCT < 0,25 ng/mL and infection is highly unlikely or - if PCT reduced > 90% of baseline value - assess AB appropriateness and/or adequacy of source control if PCT level at 48 hours is greater than 70% of baseline value | 97% < | NA | 9 (6-20) vs 11 (6-22) | ICU mortality: 10.7% vs 7.6% |
| Oliveira et al., 2013  (28 days or until death/hospital discharge) | - infection caused by Pseudomonas aeruginosa, Acinetobacter baumannii, Listeria species, Mycobacterium tuberculosis, or fungi - Staphylococcus aureus bacteremia - severe infections caused by viruses or parasites - infections that require long-term treatment (e.g. bacterial endocarditis) - localized chronic infections (e.g. chronic osteomyelitis) - more than 48 hours of AB treatment - immunosuppressed patients - patients post solid-organ transplant - patients under palliative care - patients who suffered multiple trauma, burns, or major surgery in the previous 5 days - patients diagnosed with pulmonary neoplasias, carcinoid tumors or medullary tumors of the thyroid - patients who remained in the ICU for 24 hours or less | - none | - PCT reduced > 90% of peak level (if initial value was 1 ng/mL <) or - PCT < 0.1 ng/mL (if initial value was < 1 ng/mL) - patients with decreasing SOFA score and clinical resolution of infection - maximum 7 days of AB - patients with positive blood cultures with initial SOFA score >10 - minimum 7 days of AB (regardless of PCT value) | 88 % | NA | 8.0±3.7 vs 7.2±3.5 | 28-day mortality: 32.7% vs 33.3% |
| Annane et al., 2013  (NA) | - pregnancy - burns over ≥ 15% of body surface area - trauma, outpatient or inpatient cardiac arrest, post orthopedic surgery status - drug-related neutropenia - withdrawal of life-supportive therapies or a decision to withhold them - indisputable clinical infection - antibiotic exposure ≥ 48 h during the time shortly before ICU admission | - PCT 0.5-5 ng/mL (recommended) - PCT > 5 ng/mL (strongly recommended - postoperative patients with different cut-offs (4-9 and 9 <, respectively) | - PCT <0.25 ng/mL - 0.25 ng/mL < PCT < 0.5 ng/mL (strongly encouraged) - PCT < 4 ng/mL for postoperative patients | 81%, 83%, 63% (6h, 3 days, 5 days after randomization) | NA | 5 (2-5) vs 5 (3-5) | ICU mortality: 23% vs 33% |
| Deliberato et al., 2013  (30 days) | - onset of AB therapy more than 48 hours before the date when the cultures were performed - pregnancy - infections requiring prolonged AB therapy (endocarditis, hepatic or brain abscess, deep abscess, mediastinitis, osteomyelitis - severe infection caused by viruses, parasites, fungi or mycobacteria - chronic localized infections (chronic osteomyelitis, chronic prostatitis) - patients without indication for ICU admission - negative cultures (blood, urine, tracheal aspirate or bronchoalveolar lavage fluid) in patients with suspected sepsis, severe sepsis or septic shock | - none | - PCT reduced > 90% of peak value or - PCT < 0.5 ng/mL - measure on day 0, day 5, day 7 and after that every 48 h if needed | 71% | 93% / 85% (PCT/control) | 10 [3-39] vs 11 [2-45] | ICU mortality: 2.4% vs 10.3% |
| Tang et al., 2013  (6 weeks) | - AB treatment within two weeks before recruitment - bacterial infection in other parts of body than the respiratory system - chest X-ray confirmed pneumonia - other chronic respiratory diseases - severe organ dysfunction | - PCT > 0.25 ng/mL | - none | 100% | NA | use of ABs: 46% vs 75% | hospital readmission: 5% vs 8 % |
| Layios et al., 2012  (NA) | - no informed consent - less than 48 h ICU stay | - PCT > 0.5 ng/mL (less recommended) or - PCT > 1 ng/mL (recommended) | - none | 46.3% | NA | DDD/100 ICU days: 147±206 vs 141±136 | ICU mortality: 21.7% vs 21.1% |
| Qu et al., 2012  (28 days) | - time interval between diagnosis and study inclusion > 24 hours - thyroid disease (such as thyroid adenoma) - shock (such as hypovolemic shock) - need of surgical interventions | - PCT > 0.5 ng/mL | - PCT < 0.5 ng/mL over 3 days and clinical signs and symptoms of infection improved | NA | NA | 10.9±2.9 vs 16.6±2.5 | ICU mortality: 20.0% vs 22.2% |
| Jensen et al., 2011  (60 days) | - highly elevated bilirubin levels (> 40 mg/dL) or triglycerides (> 1000 mg/dL) - pregnancy - have an ICU stay less than 24 hours - patients who were judged to be at an increased risk from blood sampling | - PCT ≥ 1 ng/mL and - broaden spectrum if PCT decrease < 10% of previous day value | - PCT < 1 ng/mL for 3 days | 82.1 % | NA | 6 (3-11) vs 4 (3-10) | 28-day mortality: 31.5% vs 32.0% |
| Long et al., 2011  (28 days) | - pregnancy - start of AB therapy >48 h before enrollment - systemic immune deficiency - withholding of life-support - active tuberculosis | - PCT > 0.25 ng/mL | - PCT < 0.25 ng/mL | NA | NA | 5 (3-6) vs 7 (5-9) | treatment success: 85.2% vs 88.9 % |
| Maravić-Stojković et al., 2011  (NA) | - redo cardiac operation - thoracic aortic surgery - active endocarditis - LVEF <30% - preoperative signs of infection (leukocyte count > 12000/L; body temperature > 38°C) | - PCT > 0.5 ng/mL - measure 24 h preoperatively; 6 h, 24 h and 48 h postoperatively | - none | NA | NA | patients on ABs: 19% vs 47% | 30-day mortality: 2.9% vs 2.9% |
| Burkhardt et al., 2010  (28 days) | - AB treatment during the previous 2 weeks - chronic liver disease - major surgery that had required hospitalization during the last 4 weeks - autoimmune or systemic disorders - dialysis - medullary C-cell carcinoma - other inflammatory diseases | - PCT > 0.25 ng/mL | - none | 87% | NA | patients on ABs: 21.5% vs 36.7% | number of days (mean) with significant health impairment: 9 vs 9 days |
| Bouadma et al., 2010  (60 days) | - pregnancy - expected stay in the ICU of less than 3 days - bone-marrow transplant or chemotherapy induced neutropenia - infections for which long-term antibiotic treatment is strongly recommended (infective endocarditis, osteoarticular infections, anterior mediastinitis after cardiac surgery, hepatic or cerebral abscesses, chronic prostatitis, or infection with Mycobacterium tuberculosis, Pneumocystis jirovecii or Toxoplasma gondii) - poor chance of survival - do not-resuscitate orders | - PCT > 0.5 ng/mL | - PCT reduced > 80% of peak value or - PCT < 0.5 ng/mL | 47% | 91% / 93 % (PCT / control group) | 6.1±6 vs 9.1±7.1 | 28-day mortality: 21.2% vs 20.4% |
| Stolz et al., 2009  (28 days) | - pregnancy - enrollment in another trial - had received immunosuppressants or long-term corticosteroid therapy - severe immunosuppression, acquired immunodeficiency syndrome - coexisting extrapulmonary infection diagnosed between day 1 and 3 requiring AB therapy for 3 days | - none | - PCT reduced > 80% of baseline value or - PCT < 0.5 ng/mL - measure 0h, 72 h then every 24 hours until day10 | NA | 86% in both groups | 10 (6-16) vs 15 (10-23) | 28-day mortality: 16% vs 24% |
| Kristoffersen et al., 2009  (NA) | - no informed consent | - PCT > 0.25 ng/mL (encouraged) or - PCT > 0.5 ng/mL (strongly encouraged) | - PCT < 0.25 ng/mL | 59% | NA | 5.1 95% CI (4.4-6) vs 6.8 95% CI (5.9-7.7) | hospital LOS ^a^  5.9 (5.1-6.9) vs 6.7 (5.9-7.7) |
| Hochreiter et al., 2009  (NA) | - lack of informed consent - AB treatment had been initiated before ICU admission - who had therapy limitations | - none | - PCT < 1 ng/mL or - PCT > 1 ng/mL but PCT reduced > 65-75 % of baseline value for 3 days | NA | NA | 5.9±1.7 vs 7.9±0.5 | in hospital mortality: 26.3% vs 26.4% |
| Schuetz et al., 2009  (30 days) | - active intravenous drug use - severe immunosuppression other than corticosteroid use - life-threatening medical comorbidities leading to possible imminent death - hospital acquired pneumonia - chronic infection necessitating AB treatment | - PCT > 0.25 ng/mL (encouraged) - PCT > 0.5 ng/mL (strongly encouraged) | - PCT < 0.25 ng/mL or - PCT reduced > 80 or 90% of peak value (strongly recommended if initial value > 10 ng/mL) | 91% | NA | 5 (1-8) vs 9 (6-11) | combined adverse outcome within 30 days: 15.4% vs 18.9% |
| Briel et al., 2008  (28 days) | - AB use within the previous 28 days - psychiatric disorders or inability to give written informed consent - not being available for follow-up - not being fluent in German - severe immunosuppression - cystic fibrosis - active tuberculosis - need for immediate hospitalization | - PCT > 0.25 ng/mL | - PCT < 0.25 ng/mL | 85% | NA | 6.2±2.5 vs. 7.1±2.2 | days with restricted activities ^a^: 8.7±3.9 vs 8.6±3.9 |
| Schroeder et al., 2009  (NA) | - lack of informed consent - already had received AB treatment prior to ICU admission | - none | - PCT < 1 ng/mL or - PCT reduced > 65-75 % of initial value over three consecutive days | NA | NA | 6.6±1.1 vs 8.3±0.7 | in hospital mortality: 21.4% vs 23.1% |
| Nobre et al., 2008  (NA) | - infections caused by Pseudomonas aeruginosa, Acinetobacter baumannii, Listeria spp., Legionella pneumophila, Pneumocystis jirovecii or Mycobacterium tuberculosis for which a prolonged duration of AB therapy is needed - severe infections due to viruses or parasites - prolonged AB therapy is needed (e.g. bacterial endocarditis, brain abscess, deep abscesses) - AB therapy started 48 hours or more before enrollment - chronic, localized infections (e.g. chronic osteomyelitis) - severely immunocompromised patients, patients on immunosuppressive therapy after solid organ transplantation - withholding of life support - absence of antimicrobial treatment despite clinical suspicion of sepsis | - none | - PCT reduced > 90% of initial value or - PCT < 0.25 ng/mL - PCT < 0.1 ng/mL on day 3 (if initial PCT < 1 ng/mL) | 81% | NA | 6.0 [3-34] vs 9.5 [2-33] | 28-day mortality: 20.5% vs 20.0% |
| Stolz et al., 2007  ( 6 months) | - other cause of symptoms than worsening of COPD - vulnerable study participants - immunosuppression - asthma - cystic fibrosis - presence of infiltrates on chest radiographs on hospital admission | - PCT > 0.25 ng/mL or - 0.1 ng/mL < PCT < 0.25 ng/mL if patient is unstable | - none | NA | NA | AB prescription: 40% vs 72% | clinical success: 82.4% vs 83.9% |
| Christ-Crain et al., 2006  (6 weeks) | - cystic fibrosis - active pulmonary tuberculosis - hospital-acquired pneumonia - severely immunocompromised patients | - PCT > 0.25 ng/mL (encouraged) or - PCT > 0.5 ng/mL (strongly encouraged) | - PCT < 0.25 ng/mL or - PCT reduced > 90% of initial value if initial PCT > 10 ng/mL | 92% | 97% in both groups | 5.8±5.3 vs 12.9±6.5 | 6 week mortality: 12% vs 13% |
| Christ-Crain et al., 2004  (10-14 days or 4-6 month (COPD)) | - severely immunocompromised patients - cystic fibrosis - active tuberculosis - nosocomial pneumonia | - PCT > 0.25 ng/mL (encouraged) or - PCT > 0.5 ng/mL (strongly encouraged) | - PCT < 0.25 ng/mL | 82.3% | NA | 10.9±3.6 vs 12.8±5.5 | mortality: 3% vs 3% |
| ^a^ presented as mean±SD, mean 95% CI (95% CI), median (IQR), median [range]; abbreviations: AB: antibiotic, ABs: antibiotics, AECOPD: acute exacerbation of chronic obstructive pulmonary disease, DDD: daily defined dose, DIC: disseminated intravascular coagulation, DNR: do not resuscitate, ED: emergency department, GCS: Glasgow Coma Scale, GP: general practitioner, HIV: human immunodeficiency virus, ICU: intensive care unit, IVDU: intravenous drug user, LOS: length of stay, LRTI: lower respiratory tract infection, LVEF: left ventricular ejection fraction, mRS: modified Rankin Scale, PCR: polymerase chain reaction, PEEP: positive end-expiratory pressure, UTI: urinary tract infection, VAP: ventilator-associated pneumonia | | | | | | | |

#
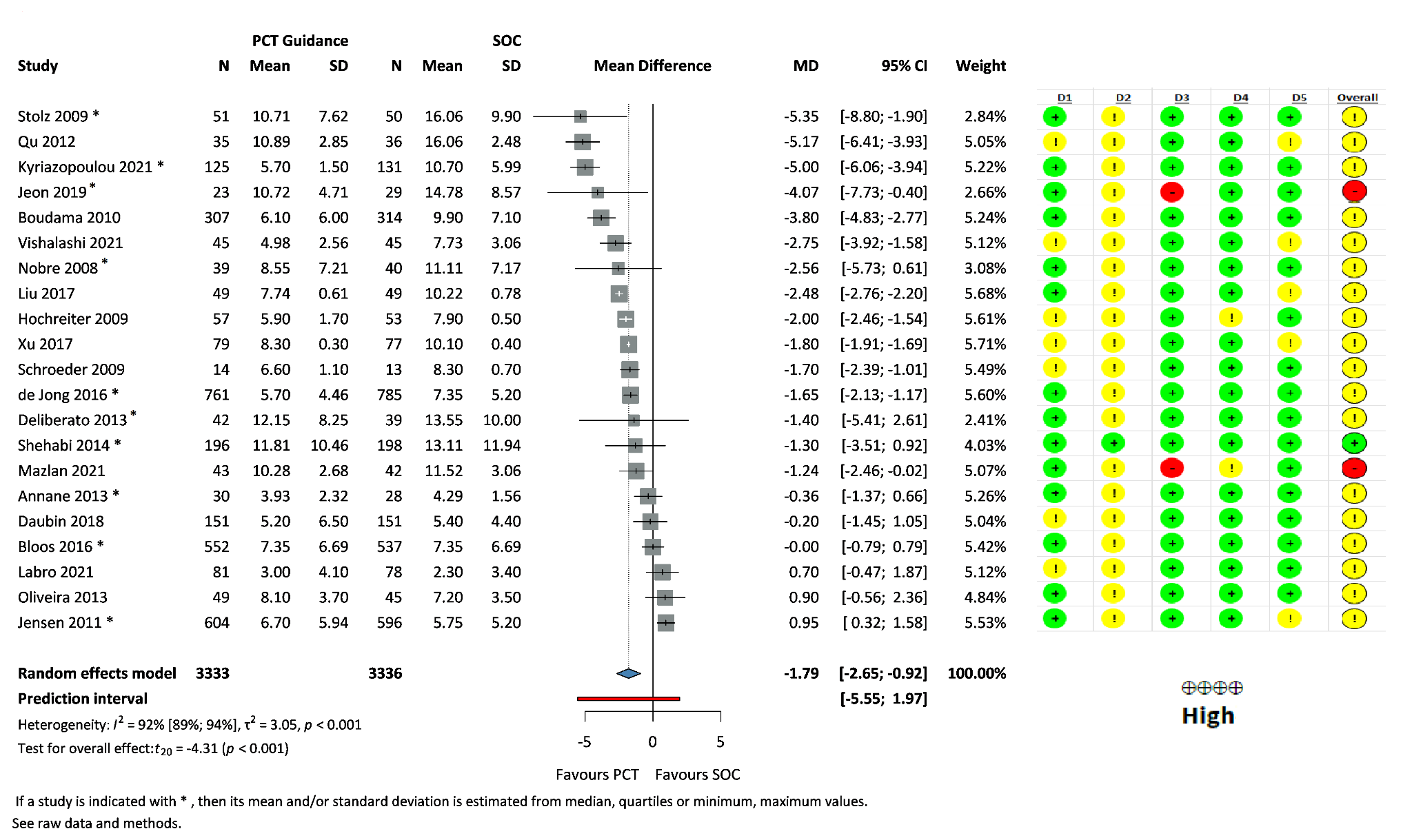
Figure S1: Forest plot of length of AB therapy

# Figure S2: Forest plot of 28-day mortality


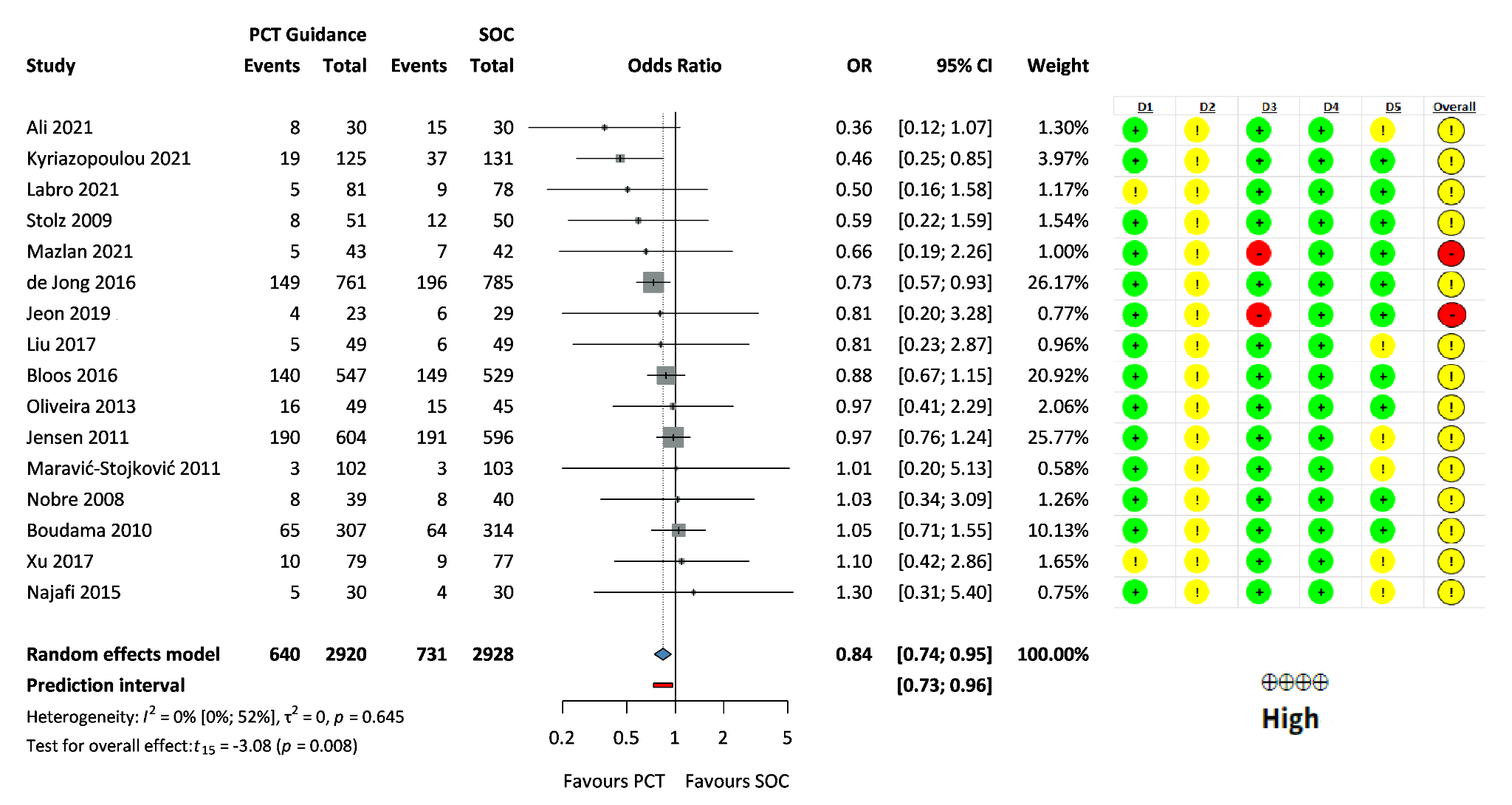


# Figure S3: Forest plot of in-hospital mortality


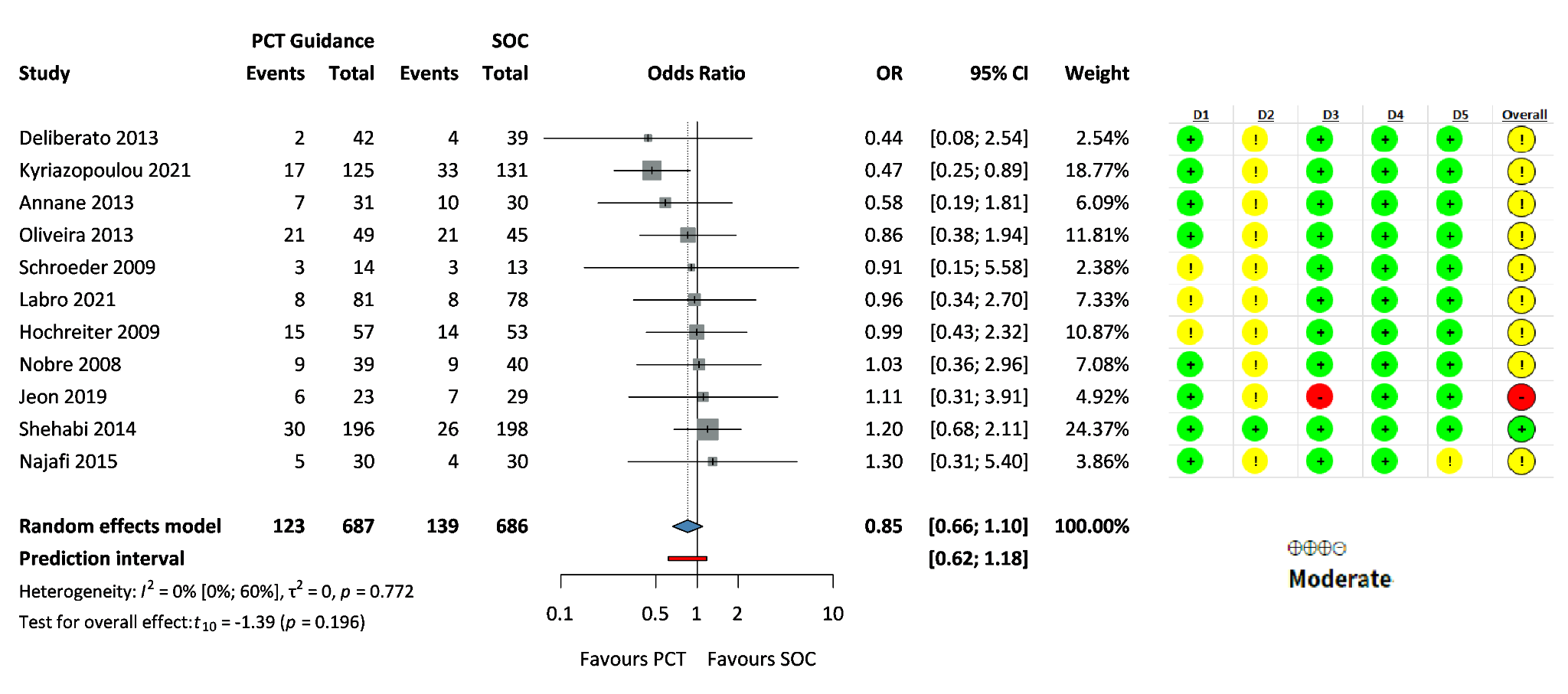


# Figure S4: Forest plot of ICU mortality


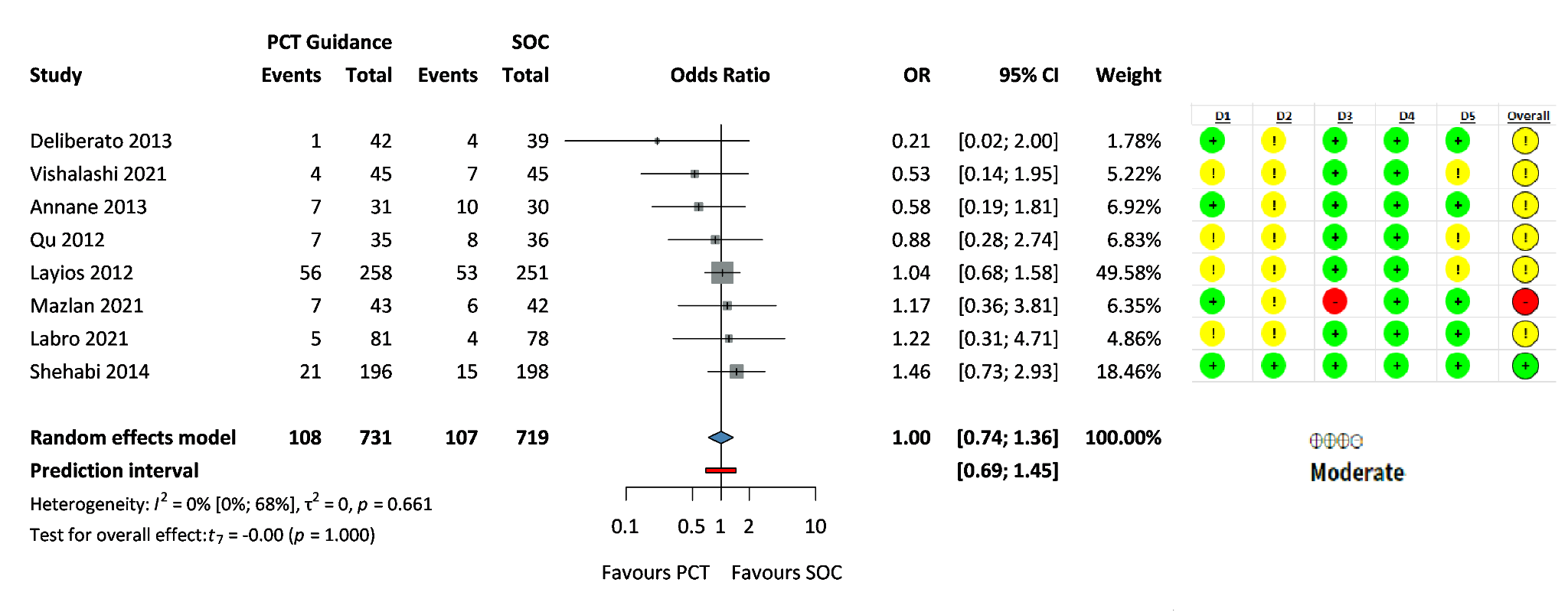


# Figure S5: Forest plot of length of ICU stay


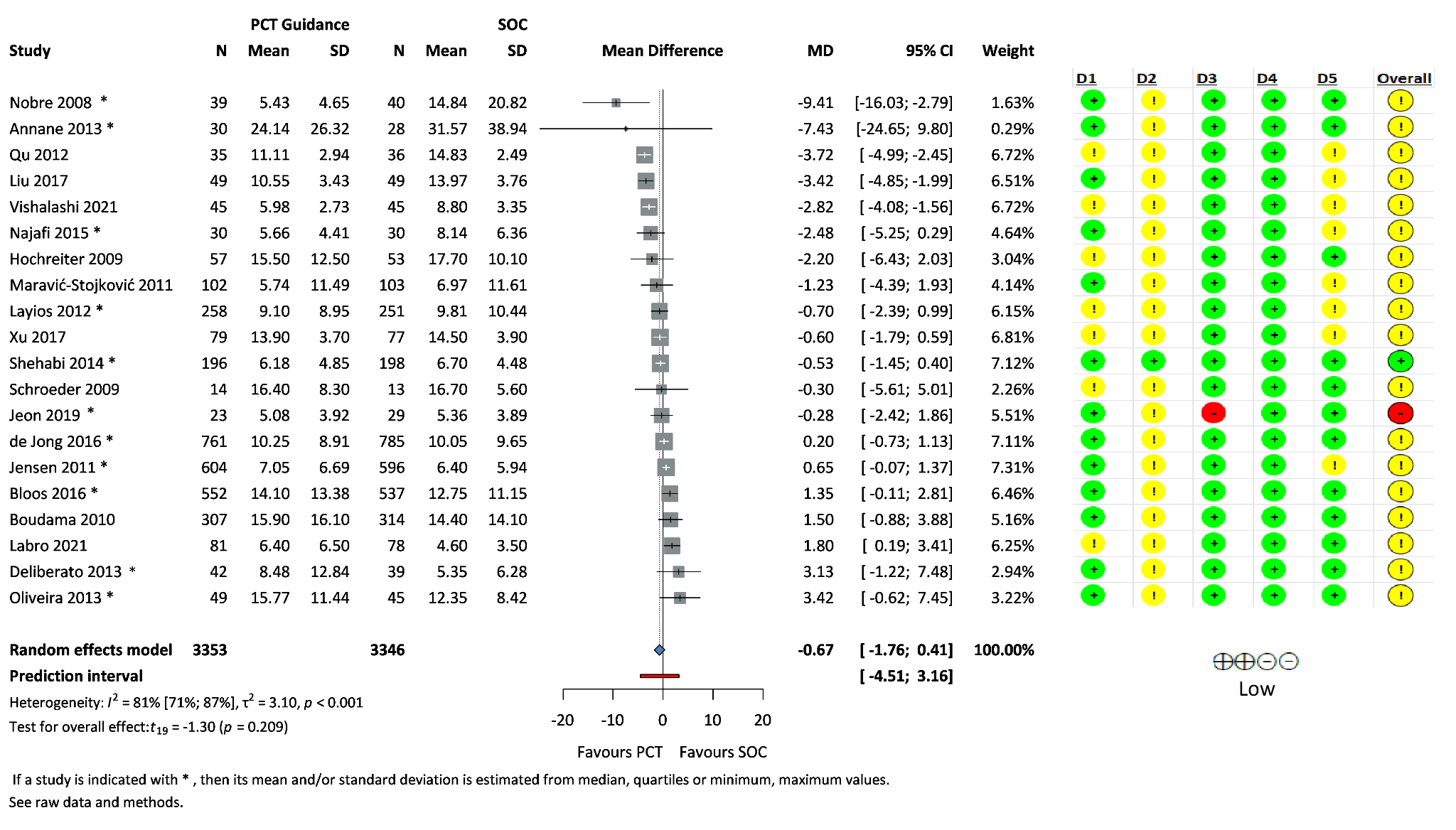


#
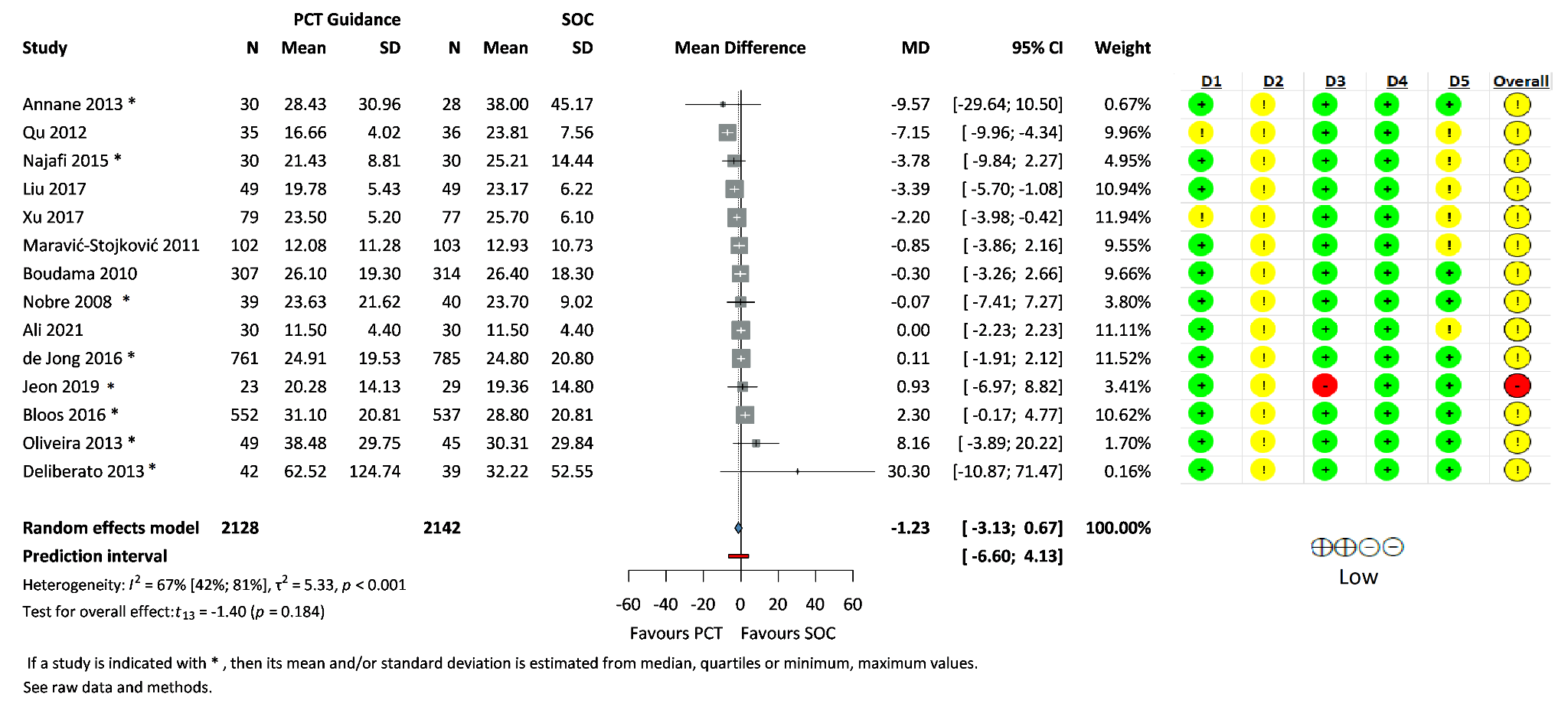
Figure S6: Forest plot of length of hospital stay

#
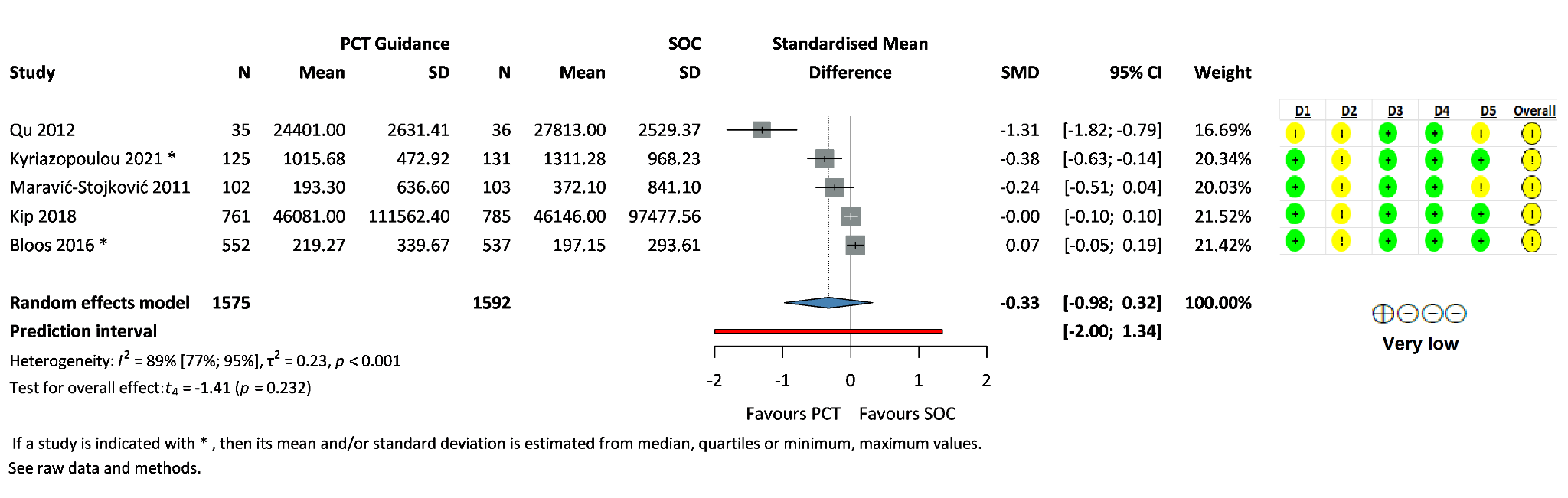
Figure S7: Forest plot of healthcare costs

# Figure S8: Funnel plots

1.
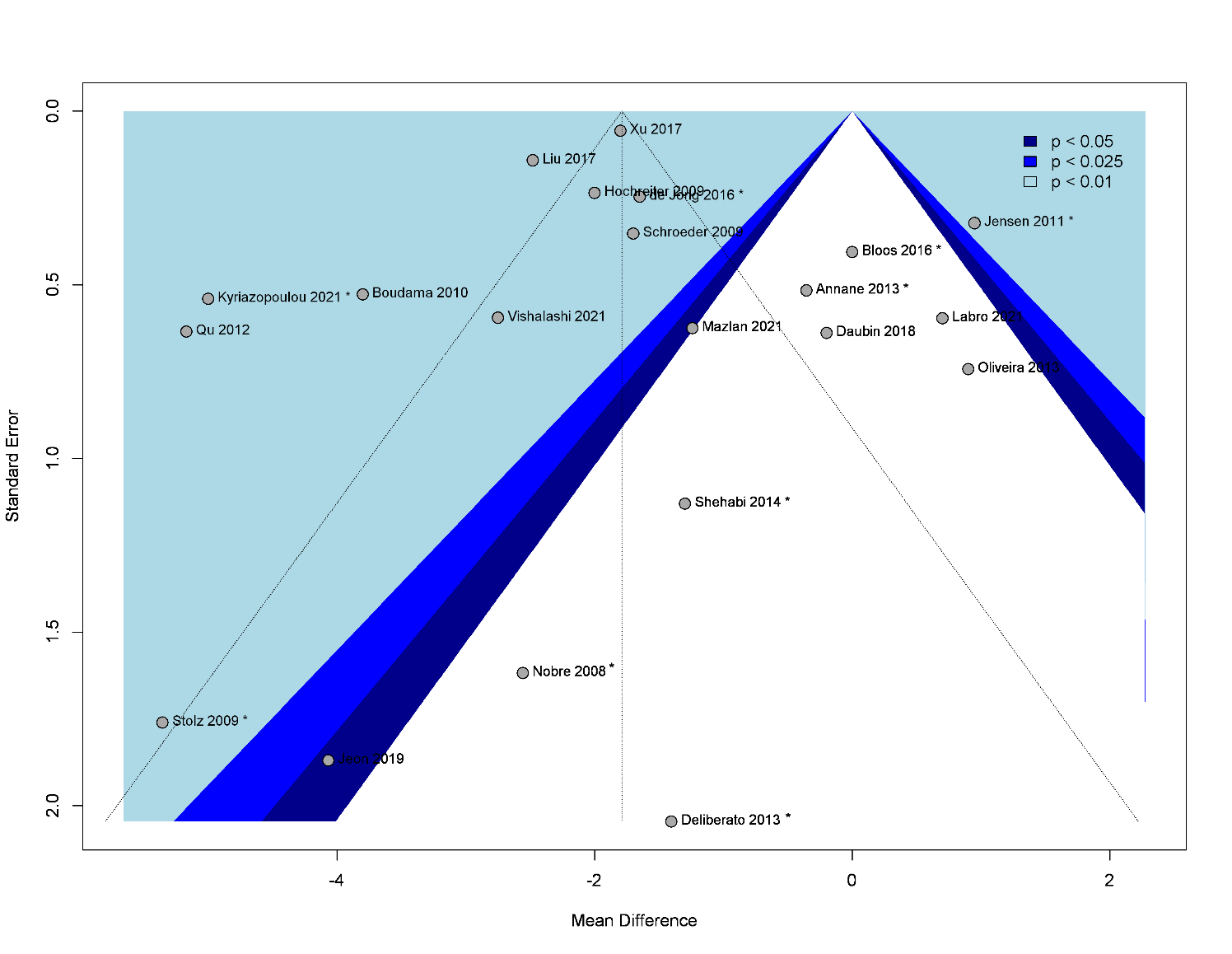
Funnel plot for length of AB therapy
2.
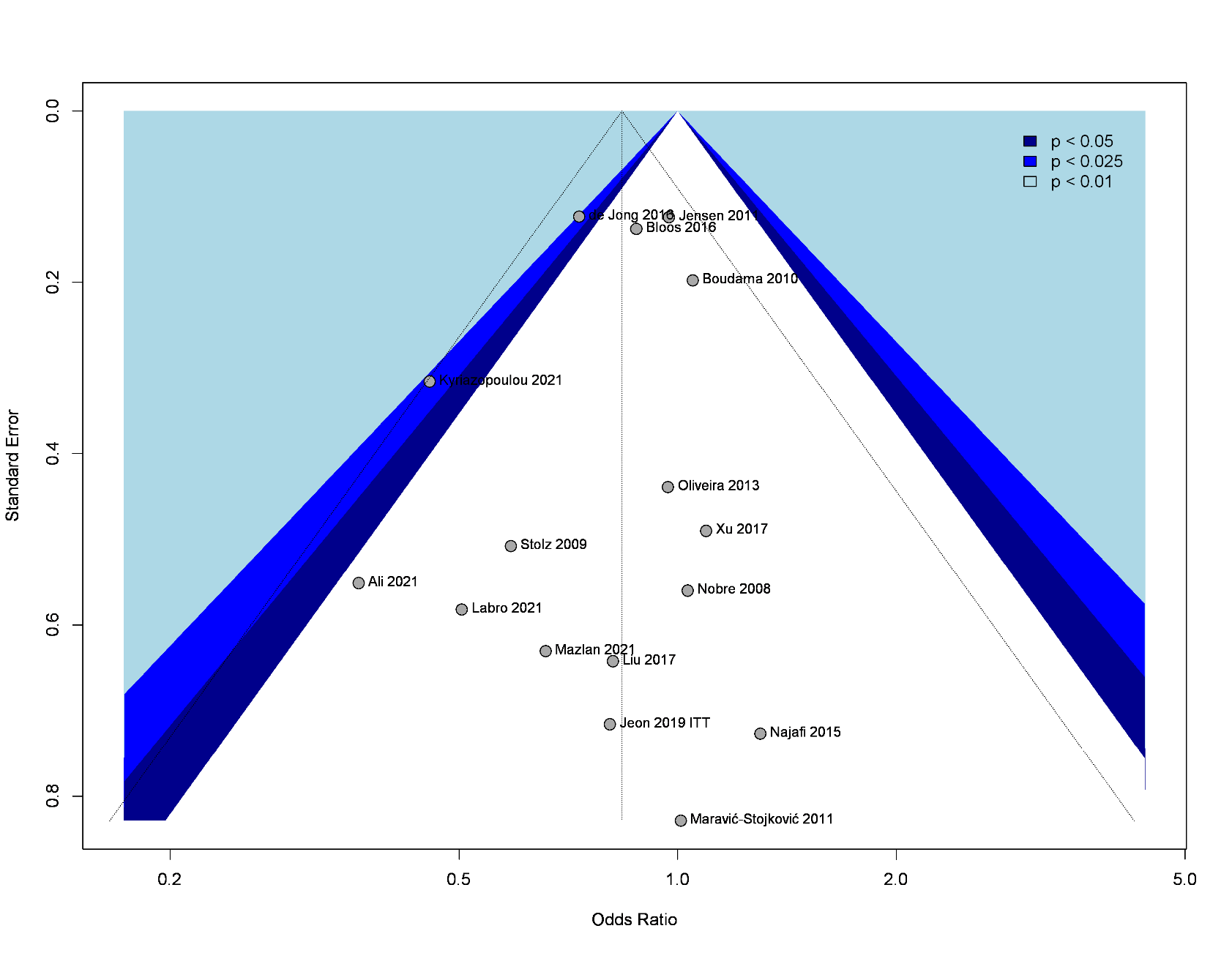
Funnel plot for 28-day mortality
3. Funnel plot for recurrent infection


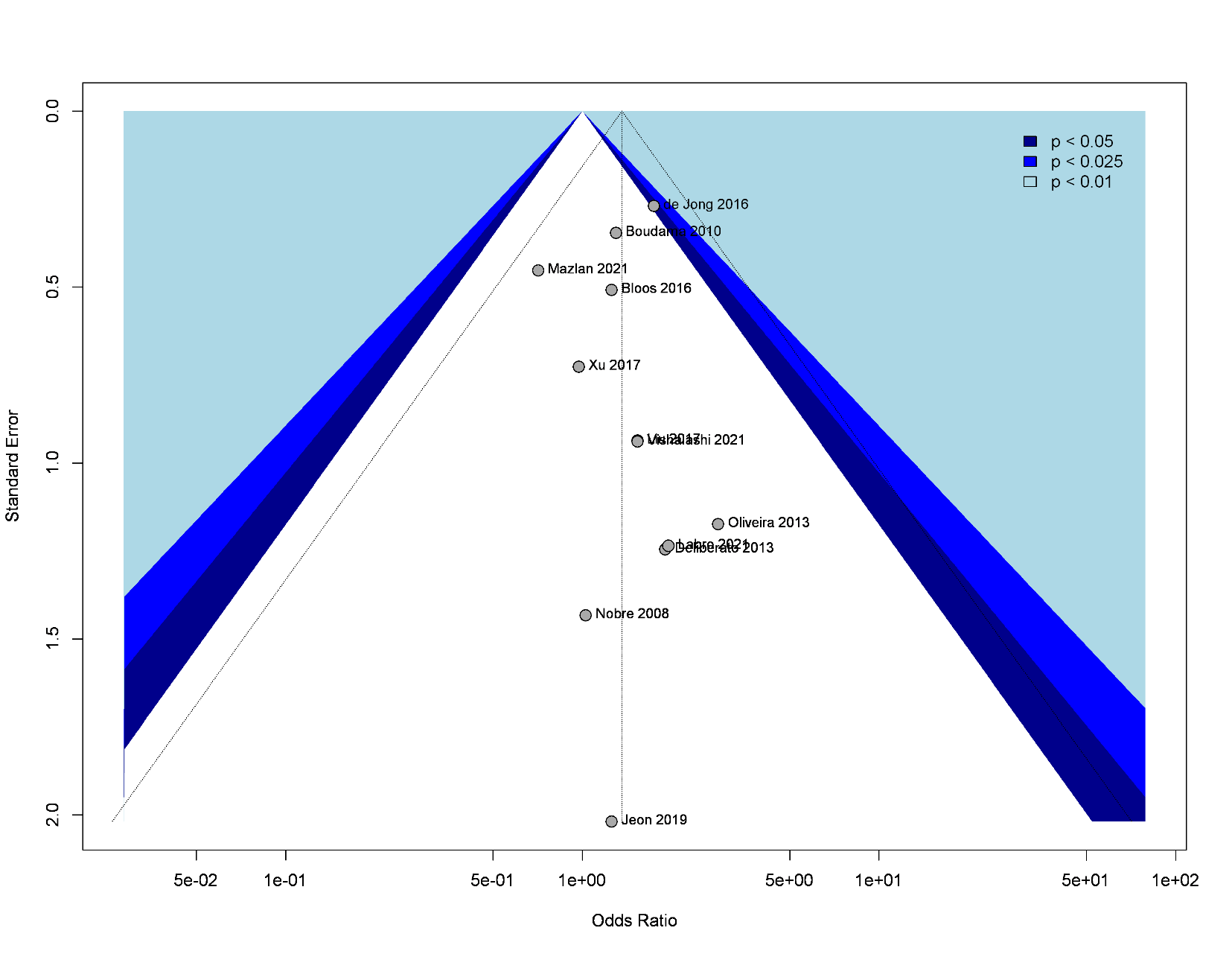


1. Funnel plot for secondary infection


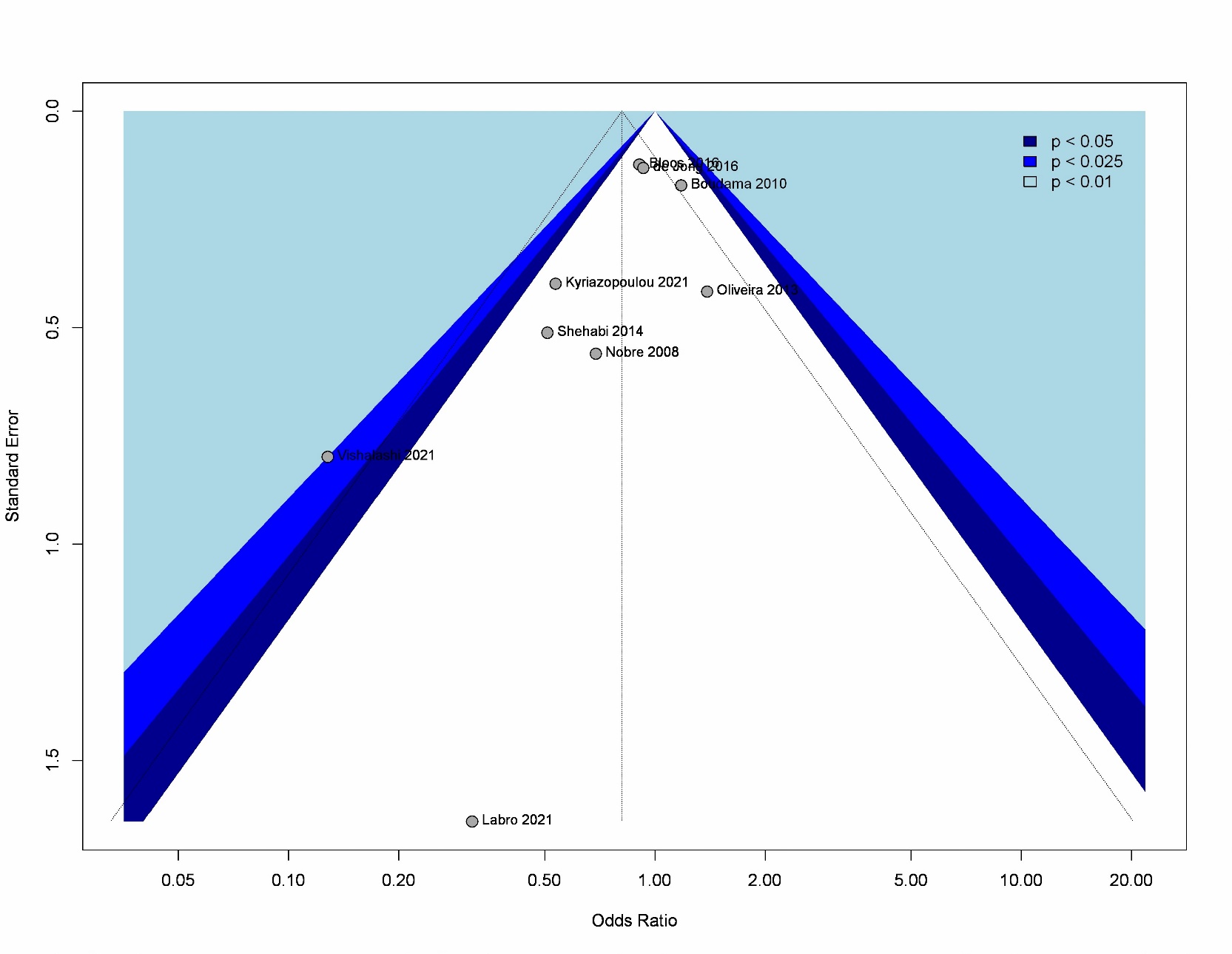


1. Funnel plot for length of ICU stay


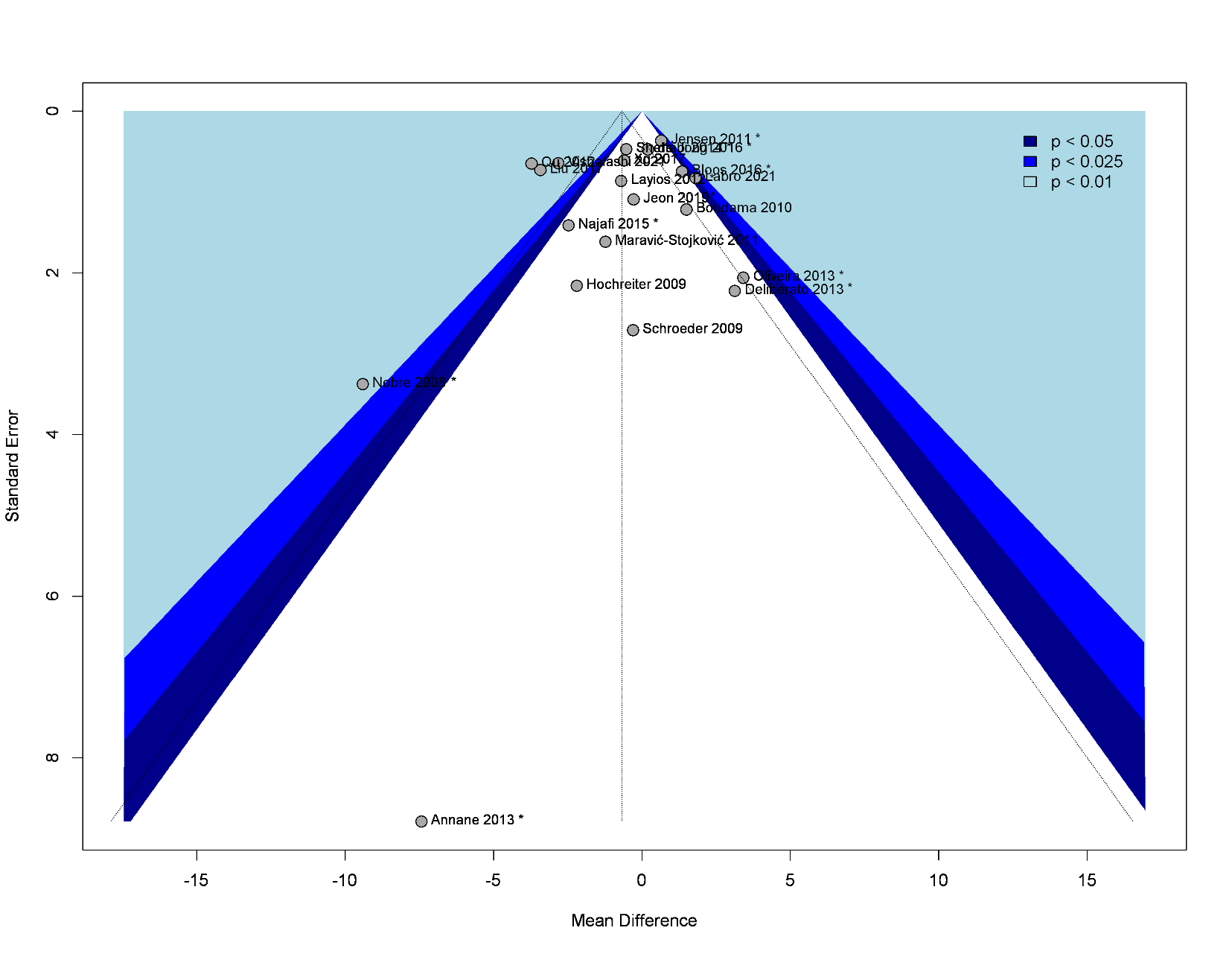


1. Funnel plot for length of hospital stay


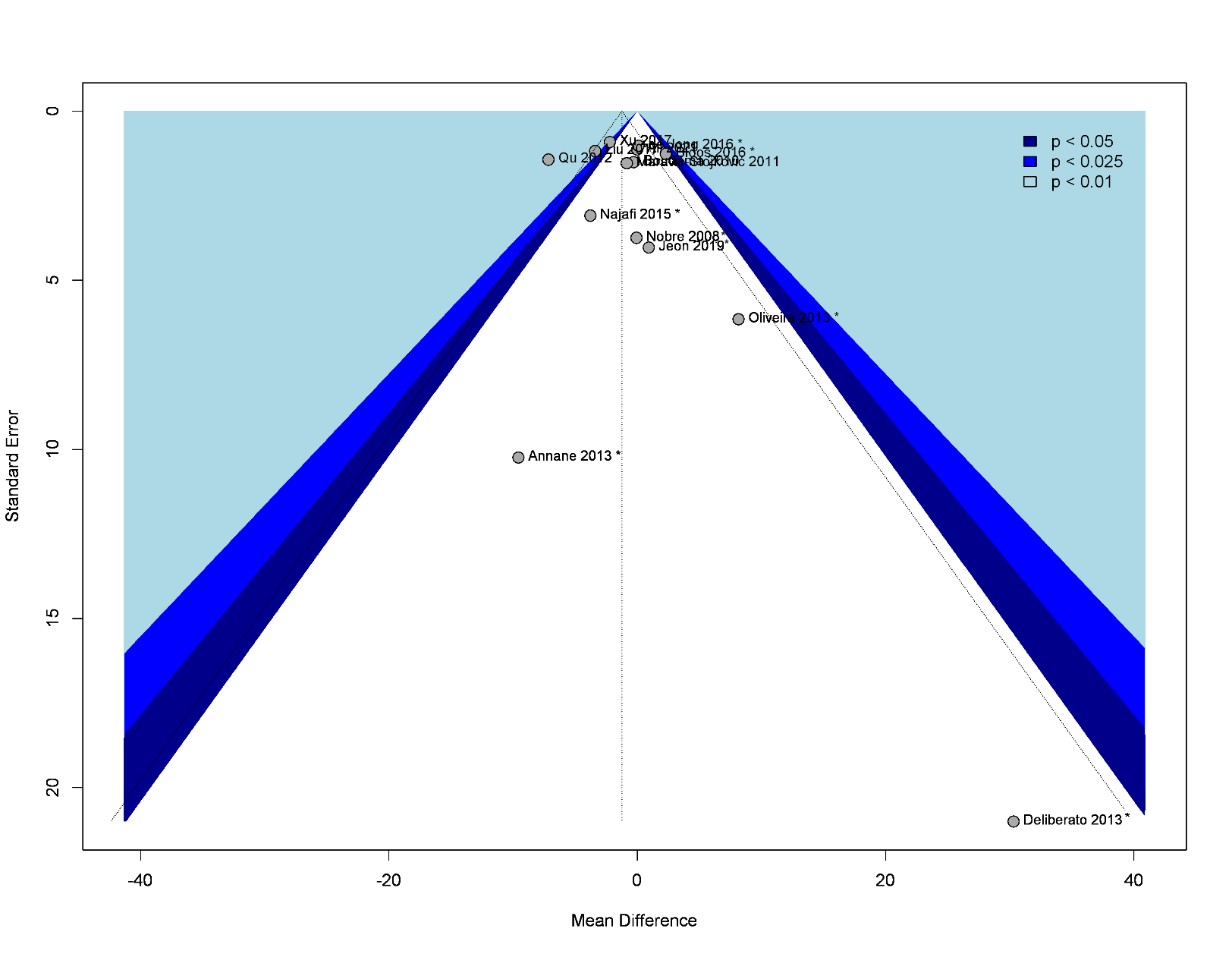

Supplement: Supplementary file 1 — Additional file1: Table S1 Prisma Checklist 2020. Table S2 Other characteristics of included studies. Figure S1 Length of AB therapy. Figure S2 28-day mortality. Figure S3 In-hospital mortality. Figure S4 ICU mortality. Figure S5 Length of ICU stay. Figure S6 Length of hospital stay. Figure S7 Healthcare costs Figure S8 (a-f) Funnel plots [file 13054_2023_4677_MOESM1_ESM.docx]
